# Beyond Glycolysis: GAPDHs Are Multi-functional Enzymes Involved in Regulation of ROS, Autophagy, and Plant Immune Responses

**DOI:** 10.1371/journal.pgen.1005199

**Published:** 2015-04-28

**Authors:** Elizabeth Henry, Nicholas Fung, Jun Liu, Georgia Drakakaki, Gitta Coaker

**Affiliations:** 1 Department of Plant Pathology, University of California Davis, Davis, California, United States of America; 2 Institute of Microbiology, Chinese Academy of Sciences, Beijing, China; 3 Department of Plant Sciences, University of California Davis, Davis, California, United States of America; Virginia Tech, UNITED STATES

## Abstract

Glyceraldehyde-3-phosphate dehydrogenase (GAPDH) is an important enzyme in energy metabolism with diverse cellular regulatory roles in vertebrates, but few reports have investigated the importance of plant GAPDH isoforms outside of their role in glycolysis. While animals possess one GAPDH isoform, plants possess multiple isoforms. In this study, cell biological and genetic approaches were used to investigate the role of GAPDHs during plant immune responses. Individual *Arabidopsis GAPDH* knockouts (KO lines) exhibited enhanced disease resistance phenotypes upon inoculation with the bacterial plant pathogen *Pseudomonas syringae* pv. *tomato*. KO lines exhibited accelerated programmed cell death and increased electrolyte leakage in response to effector triggered immunity. Furthermore, KO lines displayed increased basal ROS accumulation as visualized using the fluorescent probe H_2_DCFDA. The *gapa1-2* and *gapc1* KOs exhibited constitutive autophagy phenotypes in the absence of nutrient starvation. Due to the high sequence conservation between vertebrate and plant cytosolic GAPDH, our experiments focused on cytosolic GAPC1 cellular dynamics using a complemented GAPC1-GFP line. Confocal imaging coupled with an endocytic membrane marker (FM4-64) and endosomal trafficking inhibitors (BFA, Wortmannin) demonstrated cytosolic GAPC1 is localized to the plasma membrane and the endomembrane system, in addition to the cytosol and nucleus. After perception of bacterial flagellin, GAPC1 dynamically responded with a significant increase in size of fluorescent puncta and enhanced nuclear accumulation. Taken together, these results indicate that plant GAPDHs can affect multiple aspects of plant immunity in diverse sub-cellular compartments.

## Author Summary

Plants can be infected by all pathogen classes, significantly impacting crop production and food security. Innate immune responses are critical to plant survival but must be tightly regulated in order to avoid negative impacts on growth and development. Here, we investigated the role of glyceraldehyde 3-phosphate dehydrogenase (GAPDH) proteins in the model plant *Arabidopsis thaliana*, a mustard relative. Animals have one GAPDH isoform, which has been intensely investigated and shown to exhibit diverse moonlighting, or non-traditional, activities. Plants possess multiple GAPDH isoforms that reside in distinct sub-cellular compartments. Using a combination of genetic investigation of specific GAPDH knockouts coupled with microscopy, we found that GAPDHs regulate accumulation of reactive oxygen species and cell death in response to inoculation with the bacterial pathogen *Pseudomonas syringae*. The GAPC1 isoform exhibits diverse sub-cellular localizations and dynamically responds to perception of bacterial flagellin. The GAPC1 and GAPA1 isoforms also negatively regulate autophagy, which is an important component of plant immune responses. Taken together, our results demonstrate that multiple GAPDH isoforms act to negatively regulate plant defense responses. Negative regulators are important for precisely regulating the duration and amplitude of immune responses.

## Introduction

Innate immunity is the most ancient and evolutionarily conserved system mediating pathogen perception in animals, fungi and plants [1]. Although plants lack an adaptive immune system, germ line encoded plant immune receptors recognize pathogen derived molecules or proteins and mount a successful defense response [2]. Commonly, extracellular domains of plant immune receptors recognize conserved microbe associated molecular patterns and subsequently activate pattern triggered immunity (PTI). Primarily intracellular immune receptors recognize pathogen effectors delivered into host cells during infection resulting in effector triggered immunity (ETI) [2,3]. Both PTI and ETI result in dramatic cellular changes including the production of reactive oxygen species (ROS), Ca^2+^ influx, MAP kinase signaling, and transcriptional reprogramming [4]. Despite significant overlap in defense markers, ETI is generally viewed as a stronger response and typically culminates in a form of localized programmed cell death termed the hypersensitive response (HR) at the site of infection [5]. Consequently, constitutive activation of immune signaling can lead to seedling lethality or cell death, while insufficient activation results in enhanced susceptibility to infection [6,7]. Thus, plants have fine-tuned the duration and amplitude of immune responses at the level of the receptor and beyond to properly orchestrate plant defense responses.

Robust regulation of immune responses relies on several housekeeping proteins, including heat shock protein 90 and the ubiquitin ligase-associated protein suppressor of the G_2_ allele of *skp1* (SGT1) [8,9]. Pathogens can also target and co-opt the use of housekeeping proteins, further highlighting their importance in immune regulation [10,11]. In animals, the glycolytic housekeeping protein glyceraldehyde 3-phosphate dehydrogenase (GAPDH) contributes moonlighting activities to various alternative processes such as DNA repair, RNA binding, membrane fusion and transport, cytoskeletal dynamics, autophagy and cell death [12–14]. Due to the strong impact of GAPDHs on metabolic homeostasis and its diverse moonlighting activities, GAPDHs may be attractive targets for pathogen effectors. One example is the NleB effector, conserved in *E*. *coli* and Citrobacter rodentium, *which* O-GlcNAcylates GAPDH and disrupts GAPDH-mediated activation of transcription factors involved in regulation of innate immunity [15]. The role of metabolic checkpoints in cellular immune responses and cell death is beginning to be unraveled, revealing complex regulation by housekeeping enzymes and organelle function [16,17].

GAPDH is found in organisms from all kingdoms of life, with a high degree of sequence conservation. As a housekeeping protein GAPDH is known for its role in glycolysis, where it catalyzes the reversible conversion of glyceraldehyde 3-phosphate to 1, 3-bisphosphoglycerate [18]. Animal cells contain only one isoform of GAPDH, and many moonlighting activities as well as changes in sub-cellular localization are influenced by redox dependent post-translational modifications of GAPDH on a number of highly conserved residues [19,20]. Whether GAPDH sequence conservation carries over to regulation of its diverse functions in plants has yet to be determined. As a result of gene duplication events and diversification, plants possess multiple GAPDH isoforms [21]. Arabidopsis contains four distinct isoforms comprised of seven phosphorylating and one non-phosphorylating GAPDH. These include: chloroplastic photosynthetic GAPDHs (*GAPA1*, *GAPA2*, and *GAPB*), cytosolic glycolytic GAPDHs (*GAPC1* and *GAPC2*), plastidic glycolytic GAPDHs (*GAPCp1* and *GAPCp2*), and the NADP-dependent non-phosphorylating cytosolic GAPDH (*NP-GAPDH*) [18]. The substrate conversion by glycolytic GAPDHs catalyzes a concomitant reduction of NAD+ to NADH [22]. Arabidopsis GAPA1/2 and GAPB use NADPH to generate NADP+, which buffers free radical formation from the electron chain transport by dissipating the proton gradient at the thylakoid membrane [23,24]. Therefore, by contributing to the maintenance of the NAD(P)+ / NAD(P)H ratio of the cell, plant GAPDHs can influence both cellular redox as well as general metabolism.

All phosphorylating GAPDHs share a similar structure including a highly reactive catalytic cysteine that can undergo multiple redox-induced post-translational modifications in response to ROS and reactive nitrogen species [18]. GAPC1's catalytic cysteine residue was determined to be S-nitrosylated in Arabidopsis during ETI in two independent large-scale proteomic studies [25,26]. Hydrogen peroxide also inhibits the traditional enzymatic activity of recombinant GAPC1 and GAPA1 proteins [27–29]. In Arabidopsis, treatment with hydrogen peroxide leads to increased binding of GAPC1 with Phospholipase Dδ resulting in enhanced enzyme activity of Phospholipase Dδ [30]. Overexpression of *GAPA1* in yeast and *Arabidopsis* protoplasts inhibited ROS generation and programmed cell death induced by the apoptosis regulator BAX [31]. Treatment with cadmium or other chemicals inducing cytoplasmic oxidation leads to enhanced nuclear accumulation of Arabidopsis GAPC1 in root tip cells [32]. Thus, ROS or oxidative treatments can induce GAPDH post-translational modifications and are likely to facilitate new GAPDH protein associations, influence subcellular localization, and regulate activity in plants.

In this manuscript, we focused on the role of individual GAPDH proteins in regulating plant innate immunity using the interaction between the bacterial pathogen *P* *seudomonas* *s* *yringae* pv. *t* *omato* (*Pst*) and its plant host, *Arabidopsis thaliana* [33]. All tested individual *GAPDH* KO lines exhibited enhanced disease resistance phenotypes to both virulent and avirulent *Pst*, limiting bacterial growth and accelerating the HR. Protoplasts made from KO lines displayed increased intracellular ROS. Experiments focused on GAPC1, using a *gapc1* KO complemented with *GAPC1-GFP* driven by its native promoter. In addition to the cytosol and occasionally the nucleus, GAPC1 associated with endomembrane compartments. Perception of bacterial flagellin lead to an increase in nuclear accumulation of GAPC1-GFP as well as an increase in size of GAPC1-GFP labeled vesicles. Collectively, these data highlight plant GAPDH's involvement in diverse processes and impact on the plant innate immune response.

## Results

### Individual *GAPDH* knockouts exhibit enhanced disease resistance

To determine whether individual Arabidopsis GAPDH isoforms play a role during infection with *Pst* DC3000, we screened T-DNA insertion lines for knockout (KO) lines in distinct isoforms. KO lines were obtained for the following GAPDH isoforms: *gapa1* (At3g26650, SALK_138657 and SALK_145802), *gapc1* (At3g04120, SALK_010839), *gapc2* (At1g13440, SALK_016539), *gapCp1* (At1g79530, SAIL_390_G10 and SALK_052938), and *gapCp2* (At1g16300, SALK_137288 and SALK_008979). The SALK T-DNA KO lines for *gapa1* have not been previously published, and RT-PCR validation is provided in S1 Fig KO lines in the plastidic glycolytic *GAPDHs*, *gapCp1*and *gapCp2* were previously published [34], as were the cytosolic *GAPDHs* [22]. RT-PCR validation of *gapCp1* and *gapCp2* KOs is provided in S1 Fig Homozygous T-DNA KO lines for *GAPA2* and *GAPB* were not identified and there were no available T-DNA insertions in exons. A screen of two separate T-DNA insertion lines (SALK_023971 and SALK_067204) within the promoter of *GAPA2* yielded homozygosity for the insertion without altering gene expression. The general morphology and plant size of individual KO lines during vegetative growth resembled Col-0.

**Fig 1:**
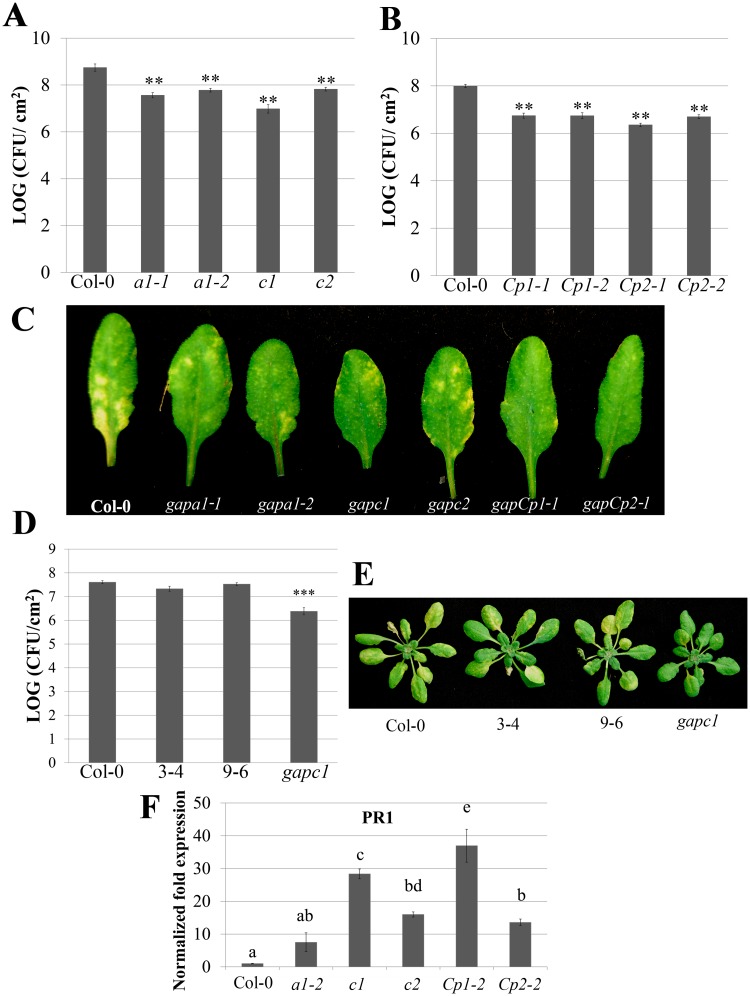
Individual *GAPDH* knockouts (KO lines) display enhanced disease resistance. **(A)** Analysis of bacterial growth in individual *GAPA1* (*a1-1*, *a1-2*), *GAPC1* (*c1*), and *GAPC2* (*c2*) KO lines illustrating bacterial population sizes four days post dip inoculation with *Pst* DC3000 at a concentration of (1×10^9^ CFU mL-1). Values represent means ± SE (n = 6). Statistical differences were detected by a two-tailed Student’s *t* test (α = 0.01) compared to wild-type Col-0. **(B)** Analyses of bacterial growth in individual *GAPCp1* (*Cp1-1*, *Cp1-2*) and *GAPCp2* (*Cp2-1*, *Cp2-2*) KO lines. Plants were inoculated and analyzed as described in (A). **(C)** Representative disease symptoms on *GAPDH* knockouts 4 days post-dip inoculation with *Pst* DC3000. **(D)** The *gapc1* KO line was complemented with native promoter (npro) driven full length *GAPC1* with a C-terminal fusion to eGFP. Two independent homozygous T3 lines, 3–4 and 9–6, complement the *gapc1* disease phenotype. Plants were inoculated and analyzed as described in (A). **(E)** Representative disease symptoms of the *gapc1* KO and *npro*::*GAPC1-GFP* complemented lines compared with Col-0 four days post dip inoculation with *Pst* DC3000. **(F)** The defense marker gene *PR1* is constitutively expressed in *GAPDH* KO lines relative to wild-type Col-0. Leaf samples taken from untreated four-week-old Col-0 and *GAPDH* KOs were used to quantify basal expression levels of *PR1*. Statistical differences were calculated using Fisher’s LSD test (α = 0.05) following a significant F-statistic. Values represent means ±SE (n = 3). Data were analyzed using the ΔΔcT method, and normalized against Arabidopsis *ELONGATION FACTOR* 1α (At5g60390).

After successfully identifying homozygous *GAPDH* KO lines, we subjected them to a variety of disease assays to determine their relative contribution during infection with *Pseudomonas syringae* pv. *tomato* (*Pst*) strain DC3000. KO lines and the Col-0 control were dip inoculated with *Pst* DC3000 and bacterial titers were determined four days post-inoculation. All of the *GAPDH* KO lines exhibited enhanced disease resistance, with a 10 fold reduction in bacterial titers compared to the Col-0 control when inoculated with virulent bacteria (Fig 1A and 1B). Lower bacterial titers correlated with a similar reduction in disease symptoms (Fig 1C). Due to the high degree of sequence conservation between human *GAPDH* with Arabidopsis *GAPC1* (68% amino acid similarity), we were particularly interested in the role of GAPC1 in the innate immune response. The *gapc1* KO line was complemented with *GAPC1-GFP* under control of its endogenous promoter (*npro*::*GAPC1-GFP*). Two independent, single insertion T3 homozygous lines were identified expressing GAPC1-GFP (S2 Fig) and bacterial growth was analyzed. Both *npro*::*GAPC1-GFP* lines 3–4 and 9–6 complemented the *gapc1* KO and exhibited similar bacterial growth and disease symptoms as wild-type Col-0 (Fig 1D and 1E). We did not observe any morphological defects in the *npro*::*GAPC1-GFP* lines (Fig 1E).

**Fig 2:**
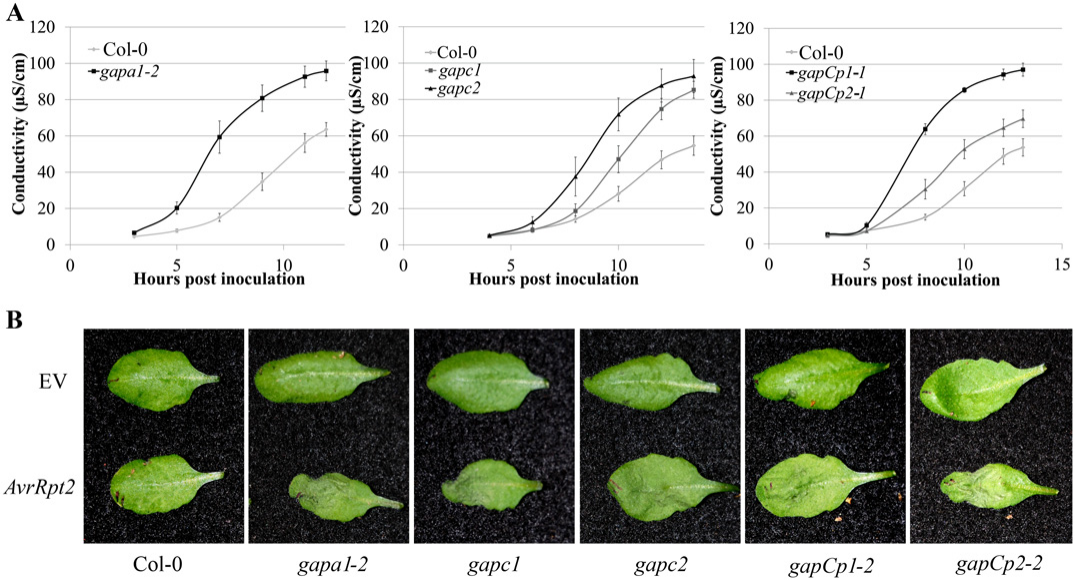
*GAPDH* knockouts displayed an accelerated hypersensitive response (HR) during effector-triggered immunity. **(A)** Electrolyte leakage measurements of individual *GAPA1-2*, *GAPC1*, *GAPC2*, *GAPCp1-1* and *GAPCp2-1* KO lines. Four-week-old leaves were infiltrated with *Pst* DC3000 *AvrRpt2* at a concentration of 4×10^7^ CFU mL-1. Values represent means ±SE (n = 4). **(B)** Macroscopic HR phenotype of Col-0 and *GAPDH* knockouts 10h post syringe infiltration with *Pst* DC3000 *AvrRpt2*. DC3000 empty vector (EV) control is shown as the top leaf. Bacterial concentrations were the same as (A).

Localized programmed cell death is a hallmark of ETI and is termed the hypersensitive response (HR). The HR can be visualized macroscopically when avirulent bacteria are syringe infiltrated into leaves at high concentrations (4×10^7^ CFU mL^-1^). *Pst* DC3000 expressing the *AvrRpt2* effector, which is recognized by the Arabidopsis RPS2 immune receptor [35], was used to investigate HR responses in individual *GAPDH* KO lines. The progression of cell death was quantified by measuring electrolyte leakage using a conductivity meter. All KO lines exhibited an accelerated HR and enhanced electrolyte leakage compared to the wild-type Col-0 control, indicating a more rapid cell death progression in the KO lines (Fig 2A). Macroscopic HR was evaluated as well, and KO lines displayed more rapid tissue collapse starting at 10h post-infiltration while Col-0 collapsed at 12h post-infiltration (Fig 2B). An enhanced disease resistance phenotype was also found after inoculation with avirulent *Pst* DC3000 expressing *AvrRpt2* (S3 Fig). Although all KO lines tested exhibit enhanced disease resistance to *Pst* DC3000, the magnitude of responses varied between individual lines. The *gapCp*s exhibited the highest resistance to virulent bacterial growth and displayed very few chlorotic symptoms, followed by *gapc1* in overall symptom reduction and bacterial growth (Fig 1A, 1B, and 1C).

We used quantitative PCR to determine if the cause of enhanced disease resistance in the *GAPDH* KOs was due to transcriptional “priming” for a defense response. *Pathogenesis-related 1* (*PR1*) is a commonly used defense marker gene whose expression is induced in response to pathogen perception and salicylic acid [36]. In unchallenged *GAPDH* KO lines, basal *PR1* expression was constitutively up-regulated compared to Col-0 (Fig 1F). This indicates that *GAPDH* KO lines may be primed for pathogen defense responses in the absence of an elicitor, leading to accelerated defense responses upon pathogen inoculation. Taken together, these results indicate that multiple GAPDH isoforms act as negative regulators of plant immune responses.

### Single *GAPDH* KO lines exhibit alterations in GAPDH enzymatic activity and transcription of multiple *GAPDH* isoforms

**Fig 3:**
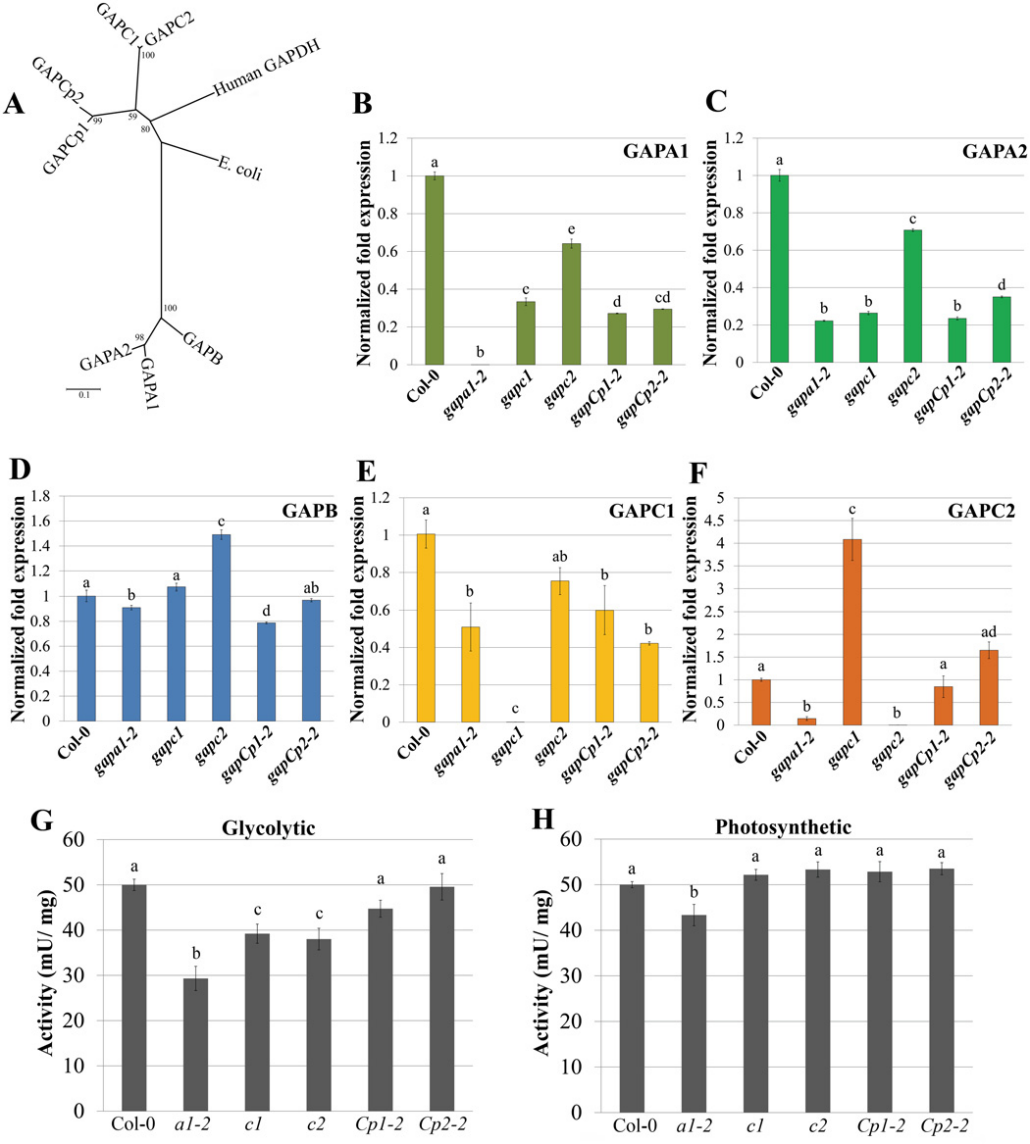
Transcription of *GAPDH* family members and enzymatic activity are altered in individual *GAPDH* knockout lines. (**A)** Unrooted phylogeny of Arabidopsis phosphorylating GAPDHs as well as human and *E*. *coli* GAPDH. Arabidopsis GAPCs and GAPCps cluster with human GAPDH, while photosynthetic GAPDHs cluster separately. Values at nodes indicate bootstrap values. Scale bar indicates the number of amino acid substitutions per site visualized in the branch length. **(B-F)** Quantitative PCR (qPCR) analyses of leaves from four-week-old plants were used to quantify basal expression levels of phosphorylating GAPDHs across different KO lines. Expression values are shown relative to wild-type Col-0. Data were analyzed using the ΔΔcT method, and normalized against Arabidopsis *ELONGATION FACTOR* 1α (At5g60390). Statistical differences were calculated using Fisher’s LSD test (α = 0.05) following a significant F-statistic. Values represent means ±SE (n = 3). **(G)** Leaf protein extracts from four-week-old *GAPDH* KO lines and Col-0 were assayed for GAPDH activity in the glycolytic direction. GAPDH activity (mU/mg total protein) in the single KO lines was assayed at 340nm for the reduction in NAD+. Data from a minimum of 5 independent runs were normalized to Col-0 and combined for statistical analyses. Statistical differences were calculated using Fisher’s LSD test (α = 0.05) following a significant F-statistic. Values represent means ±SE (n≥9). **(H)** Leaf protein extracts from four-week-old *GAPDH* KO lines and Col-0 were assayed for GAPDH activity in the Calvin cycle direction (photosynthetic). The GAPDH activity in the photosynthetic reaction was measured as described in (G) as the reduction in NADP+ over time. Statistical analyses were performed as described in (G).

Plant GAPDH isoforms arose through multiple gene duplication events [21]. Phylogenetic analyses of the seven Arabidopsis GAPDH isoforms in addition to human and *E*. *coli* GAPDH reveals a high degree of sequence conservation (Fig 3A) [37]. Gene duplication can lead to diversification of biochemical functions and expression patterns. However, duplicated genes may also carry out similar or overlapping functions making genetic analyses challenging. Previously, it was reported that a KO in the non-phosphorylating *GAPDH* induced higher level expression of *GAPC1* [38]. In order to investigate if single *GAPDH* KOs induce differential regulation of additional isoforms, we performed quantitative real-time PCR (qPCR) analyses to investigate basal expression of *GAPDHs* on four-week-old plants. Both *GAPCp1* and *GAPCp2* were expressed at a very low level and were excluded from the analyses based on their high Cq values (Cq = 34). qPCR analyses of the individual KO lines revealed complex transcriptional regulation of some *GAPDH* family members relative to wild-type Col-0 (Fig 3B–3F). *GAPA1* and *GAPA2* were down-regulated in all the *GAPDH* KOs, while *GAPB* expression was unchanged in the majority of lines (Fig 3B, 3C, and 3D). Both *GAPC1* and *GAPC2* were down-regulated in *gapa1-2* (Fig 3E and 3F). *GAPC2* expression was up-regulated in the *gapc1* line, presumably to help compensate for the loss of *GAPC1;* however, *GAPC1* expression was not significantly different from Col-0 in the *gapc2* line. Overall, *GAPA1*, *GAPA2* and *GAPC1* had the most significant alterations in expression levels in the *GAPDH* KO lines. Thus, *GAPDH* family members appear to be under complex regulation, with epistatic interactions occurring between *GAPA1* and the cytosolic isoforms.

To assess Arabidopsis GAPDH enzymatic activity in individual KO lines, whole-leaf homogenates were used to analyze rates of glycolysis and the Calvin cycle. While cytosolic GAPC1 and GAPC2 have conserved sequence and glycolytic function with their animal and yeast counterparts, plants have evolved chloroplastic GAPDHs that function in the Calvin cycle. The basic biochemical reaction performed by GAPDH isoforms in glycolysis and the Calvin cycle is the same, with the direction of the reaction being reversed. The direction being assayed can be controlled for *in vitro* by utilizing a two-step enzymatic assay starting with reagents that preferentially drive substrate production in either direction. We used aldolase and fructose 1, 6-bisphosphate or 3-phosphoglycerate (with endogenous phosphoglycerate kinase) to assess GAPDH enzymatic activity in the direction of either glycolysis (Fig 3G) or the Calvin cycle (Fig 3H), respectively. Only *gapa1-2* exhibited significantly impaired activity in both directions. This could be explained by the decreased transcript abundance of the cytosolic GAPDH isoforms (*GAPC1* and *GAPC2*) in addition to chloroplastic *GAPA2* in *gapa1-2*. Both *gapc1* and *gapc2* exhibited significantly reduced activity in the glycolytic direction. The *gapCp* lines were not altered in activity as compared to Col-0. It is possible that GAPDH activity is reduced in plastids of *gapCp* KO lines, but this decrease is below the level of detection using a whole leaf assay. Chloroplasts play a role in the initiation and propagation of the HR, the generation of ROS involved in transcriptional reprogramming of defense-related genes, and limiting cell death [39,40]. Therefore, changes in plastid GAPDH activity may alter chloroplast contributions to immunity.

### GAPDH enzymatic activity and transcription of distinct *GAPDH* isoforms dynamically changes during the immune response

**Fig 4:**
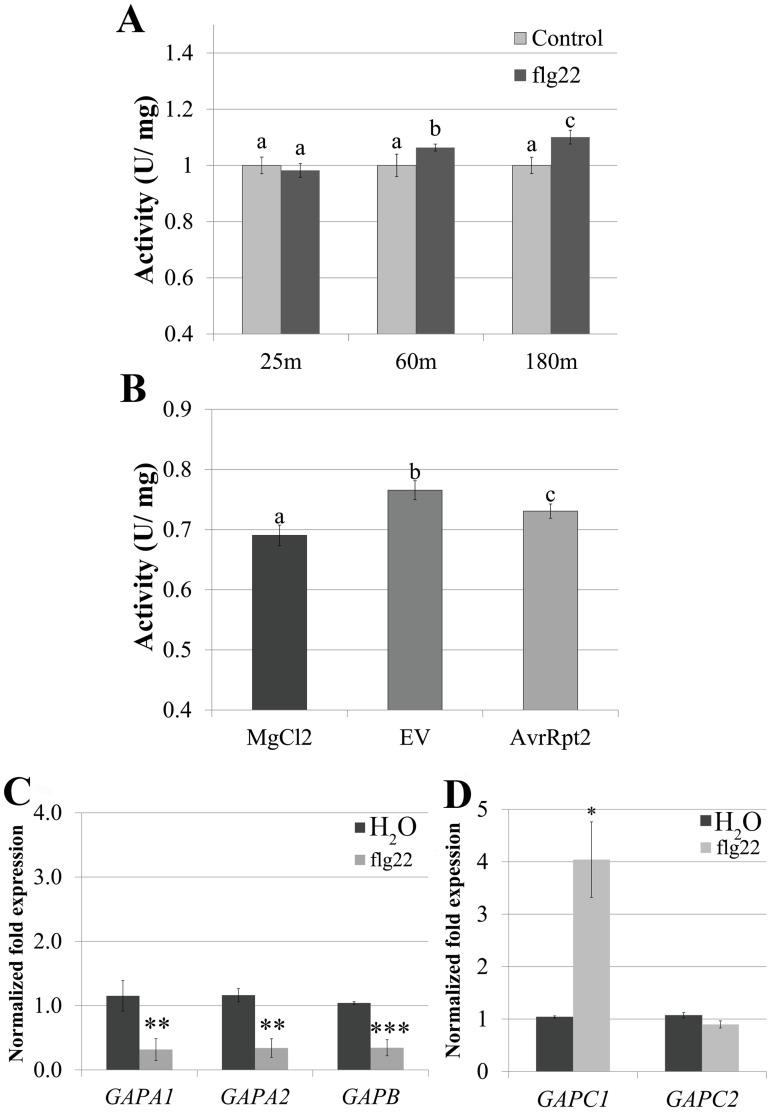
*GAPDH* transcription and enzymatic activity dynamically change during immune responses. (**A)** GAPDH glycolytic activity assays were performed on four-week-old Col-0 after syringe infiltration with 5μM flg22 or H_2_O at 25 min, 60 min, and 180 min post-infiltration. Statistical differences were detected by a two-tailed Student’s *t* test (α = 0.01) compared to water-treated samples at each time point. **(B)** Col-0 was infiltrated with 4×10^7^ CFU mL-1 *Pst* DC3000 *AvrRpt2*, DC3000 empty vector (EV) or MgCl_2_. Samples were harvested for the glycolytic activity assay 8h post-infiltration. Statistical analyses were performed as described in (A). **(C-D)** Quantitative PCR (qPCR) analyses of four-week-old Col-0 leaves 3h post-infiltration with 5μM flg22 or water. **(C)** and **(D)** segregate separate qPCR runs. Values represent means ± SE (n = 3). Statistical differences were detected by a two-tailed Student’s *t* test (α = 0.01, and 0.001) compared to H_2_0 treated controls. Data were analyzed using the ΔΔcT method, and normalized against Arabidopsis *ELONGATION FACTOR* 1α (At5g60390).

GAPDH enzymatically catalyzes the only reductive step in glycolysis and the Calvin cycle, and has been linked to programmed cell death in animal systems [14,41]. Since changes in GAPDH activity have been linked to cell death phenotypes in other organisms, we examined changes in GAPDH enzymatic activity during innate immune responses. Plant immune responses were evaluated after activation of Arabidopsis FLAGELLIN SENSING2 (FLS2), a pattern-triggered immune receptor which detects a 22 amino acid epitope of bacterial flagellin termed flg22 [42]. Four-week-old Arabidopsis plants were infiltrated with either 5μM flg22 or 10mM MgCl_2_. Glycolytic GAPDH activity was evaluated in samples harvested at 25min, 1h and 3h post-infiltration. Enzymatic activity significantly increased in flg22 infiltrated samples taken at 1h (p < 0.05) and 3h (p < 0.01) compared with MgCl_2_ infiltrated samples (Fig 4A).

To determine whether transcriptional regulation of GAPDHs occurs during PTI, we performed qPCR analyses using five of the seven phosphorylating GAPDH genes in the Arabidopsis Col-0 ecotype. Four-week-old Col-0 plants were infiltrated with either 5μM flg22 or water and leaf tissue was harvested 3h post infiltration. At 3h post infiltration *GAPA1*, *GAPA2* and *GAPB* transcripts were slightly down-regulated to less than half the control, while *GAPC1* transcript levels increased by more than two-fold (Fig 4C and 4D). These results demonstrate contrasting regulation of photosynthetic and glycolytic *GAPDHs* during PTI. GAPDH enzymatic activity increased in the glycolytic direction during PTI (Fig 4A), consistent with an increase in transcription of *GAPC1*. In order to evaluate alterations in glycolytic GAPDH enzymatic activity during ETI responses, four-week-old Col-0 plants were infiltrated with 10mM MgCl_2_ or a bacterial suspension of *Pst* DC3000 carrying empty vector or *AvrRpt2*. Tissue was harvested at the first signs of the HR when vein silvering was initially visible (~8h post-infiltration). GAPDH activity was significantly increased in leaves infiltrated with *Pst* DC3000 empty vector compared to MgCl_2_, as well as in leaves undergoing ETI responses (Fig 4B). We were unable to detect a gross change in total GAPDH protein levels during bacterial infection or flg22 elicited immune responses using anti-GAPDH western blotting (S4 Fig). However, the sensitivity of western blotting is antibody dependent [43]. Therefore, our antibody may not be sensitive enough to detect minor changes in protein abundance.

### Intracellular ROS is enhanced in *GAPDH* knockout lines and all phosphorylating GAPDH proteins are redox sensitive

All *GAPDH* KO lines exhibited enhanced disease resistance to virulent and avirulent *Pst* DC3000. One potent set of anti-microbial molecules produced by plant cells during the innate immune response are reactive oxygen species (ROS). It is well documented that the role of extracellular ROS production mediated by the NADPH oxidase respiratory burst oxidase-D (RBOHD) is critical to mounting an effective defense response to bacterial pathogens [44]. A luminol-based extracellular ROS assay using flg22 as an elicitor was not able to cause a significant alteration in extracellular ROS production for *gapc1* and *gapc2* mutant lines compared to Col-0. Interestingly, when all ROS data was analyzed together, *gapa1-2* had a significantly reduced burst compared to Col-0 (S5 Fig). GAPA1 is localized to the chloroplasts, a site of significant intracellular ROS production [40]. It is possible that loss of GAPA1 alters redox homeostasis, dampening the ROS burst produced by the NADPH oxidase RBOHD.

**Fig 5:**
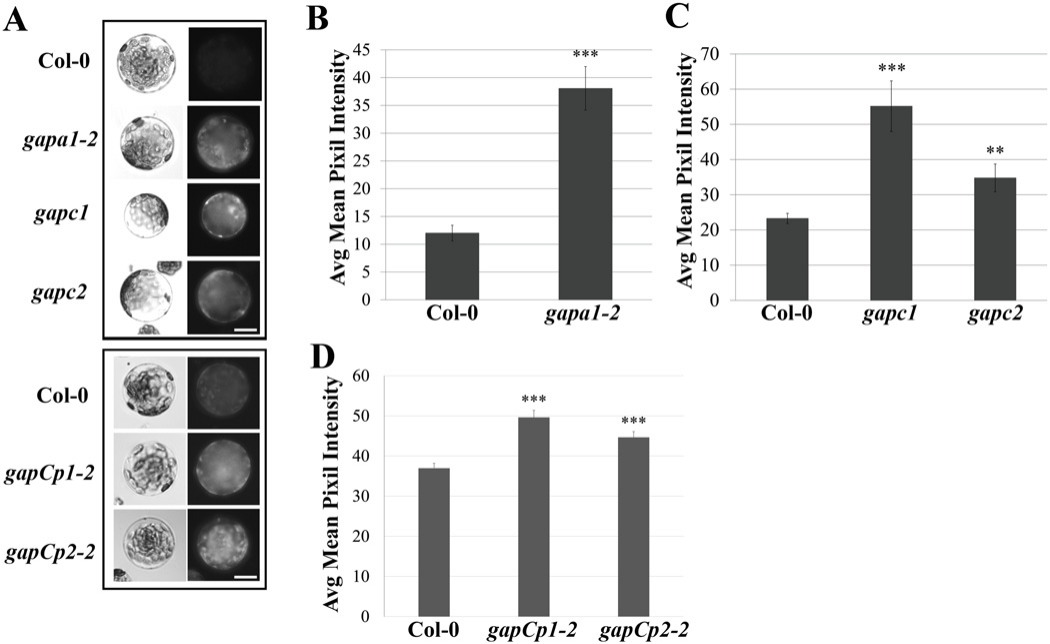
*GAPDH* knockout lines exhibit an increase in basal intracellular ROS. **(A)** Histochemical detection of ROS in protoplasts isolated from the indicated genotypes upon incubation with the fluorescent probe H_2_-DCFDA. Protoplasts were left under bright light for 1h and 750 nM of H_2_-DCFDA was added 15 min prior to imaging. Protoplasts were imaged using an epifluorescence microscope fitted with a GFP filter. Boxes indicate separate experiments and bar = 10 μm. **(B-D)** Quantification of H_2_-DCFDA fluorescence. Pixel intensity corresponding to fluorescence of intact protoplasts was quantified across three experimental replicates (n≥25) and combined for statistical analyses. Statistical differences were detected by a two-tailed Student’s *t* test (α = 0.01** and = 0.001***).

GAPDHs can directly impact cellular redox potential through their involvement in the reducing step of either glycolysis or the Calvin cycle [18,24]. Therefore, the basal intracellular ROS levels of *GAPDH* KO lines were analyzed. Protoplasts were isolated from four-week-old Col-0, *gapa1-2*, *gapc1*, *gapc2*, *gapCp1-2* and *gapCp2-2* plants and incubated under bright light for 1h since chloroplastic isoforms are light activated enzymes [21,23]. Following incubation in the light, the intracellular ROS probe H_2_DCFDA was added and protoplasts were kept in the dark for 15 min prior to imaging (Fig 5A). The extent of H_2_DCFDA fluorescence was quantified from the pixel intensity for each genotype (Fig 5B, 5C, and 5D). Although all *GAPDH* KO lines exhibited significantly enhanced basal intracellular ROS (p<0.01), the magnitude of enhanced ROS varied between lines (Fig 5A–5D). The accelerated cell death observed across *GAPDH* KO lines during the HR may be explained by increased intracellular ROS production in response to bright light stimulation, as HR development depends on light and plants are placed under a light bank after inoculation [45].

Previous reports indicated that recombinant GAPA1, GAPC1 and GAPC2 are sensitive to hydrogen peroxide treatment, supporting a hypothesis for GAPDHs as cellular redox sensors [27–29]. In order to investigate if all phosphorylating GAPDH proteins are sensitive to hydrogen peroxide, recombinant proteins were purified from *E*. *coli* and their enzymatic activity assessed before and after incubation with hydrogen peroxide (S5B Fig). As previously shown, GAPC1 and GAPC2 activity was decreased after incubation with hydrogen peroxide (S5B Fig, [28,29]). Furthermore, GAPCp1 and GAPCp2 activity was also inhibited upon treatment with hydrogen peroxide (S5B Fig). At lower concentrations, ROS have been described as acting as inter- and intracellular signaling molecules which may condition cells for accelerated responses to stimuli [46]. If GAPDHs are acting as cellular redox buffers, loss of one of these isoforms may allow for greater ROS accumulation.

### GAPC1 localizes to diverse subcellular compartments, including the plasma membrane and is sensitive to endosomal trafficking inhibitors

**Fig 6:**
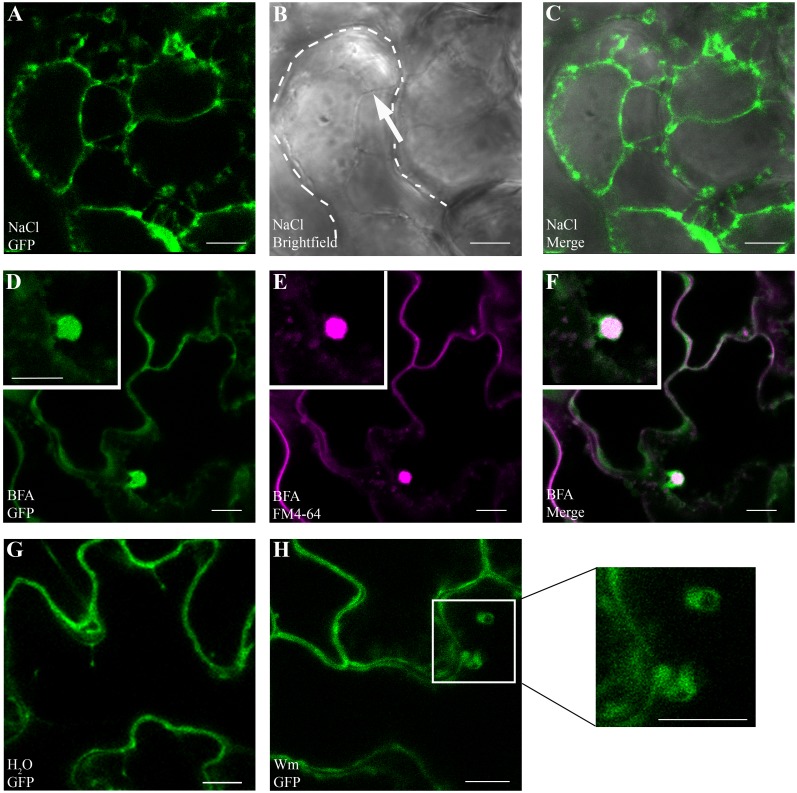
GAPC1-GFP localizes to intracellular membranes and localization is altered by endosomal trafficking inhibitors. Confocal micrographs of the *gapc1* KO complemented with *npro*::*GAPC1-GFP* (line 3–4) show optical sections of three-week-old leaves. **(A- C)** Plasmolysis using 1 M NaCl demonstrates that GAPC1-GFP is localized to Hechtian strands, providing evidence of plasma membrane localization. Arrow indicates Hechtian strand, and dotted line marks the cell boundary. **(D-F)** Leaves treated for 1.5 h with 30 μM BFA and co-stained with 1 μM FM4-64 highlight localization of GAPC1-GFP in BFA bodies. **(G-H)** Leaves treated with 33 μM Wortmannin (Wm) have an increased size of GAPC1-GFP fluorescent puncta relative to water treatment. Inset shows enlarged representative fluorescent puncta. Bar = 10 μm.

We chose to investigate GAPC1 localization in detail due to its highly conserved amino acid sequence similarity to GAPDHs in other organisms (Fig 3A). In addition to cytosolic and nuclear localizations, animal systems have linked GAPDH to endosomal movement and membrane fusion [47–49]. Signaling platforms at the plasma membrane and endomembrane are proving to be crucial in defense signaling of Arabidopsis as well as animal systems [50]. Arabidopsis GAPC1 has been reported to be primarily cytosolic, with some nuclear re-localization events in cells stressed by cadmium [32], but no endomembrane localization has been described. Our results indicating a change in glycolytic activity led us to hypothesize that GAPC1 undergoes dynamic partitioning during the immune response potentially mediated by subcellular re-localization. We investigated GAPC1 localization by confocal microscopy using the *npro*::*GAPC1-GFP* line 3–4. Plasmolysis using 1M NaCl revealed GAPC1-GFP localized to Hechtian strands, indicating partial plasma membrane localization (Fig 6A, 6B, and 6C). Western blotting after membrane fractionation in wild-type Col-0 using α-GAPDH shows endogenous GAPDH is present in nuclear, cytoplasmic and membrane fractions (S6 Fig). Thus, GAPC1 exhibits diverse subcellular localizations in the absence of stress conditions.

In order to further probe the relationship between GAPC1 and cellular membranes, we treated the first true leaves of three-week-old plants grown in soil with either 30μM Brefeldin A (BFA) or 33μM Wortmannin and examined them by confocal microscopy. BFA is known to inhibit ARF-GEFs and block the Golgi dependent secretion pathway resulting in the characteristic formation of “BFA bodies”, while Wortmannin is an inhibitor of phosphatidyl-inositol 3-kinase, and interferes with endocytosis and vacuolar sorting [50–52]. In the presence of BFA, characteristic BFA bodies stained with FM4-64 co-localized with GAPC1-GFP, indicating that GAPC1-GFP localization is BFA-sensitive (Fig 6D, 6E, and 6F). FM4-64 is an amphiphilic styryl dye that fluoresces in hydrophobic environments such as lipid membranes and is commonly used as an endocytic marker [53]. In animal systems BFA induces the ADP-ribosylation of two proteins: GAPDH and CtBP3/BARS [54,55]. However, subcellular localization of GAPDH in response to BFA treatment has not been previously visualized in either plants or animals. In the Wortmannin treated seedlings, an increase in the size of endosomes occurred (Fig 6G and 6H). These data indicate that GAPC1-GFP localization is sensitive to the inhibition of Golgi-mediated and late endocytic trafficking pathways. Furthermore, these results highlight the diverse sub-cellular localization of GAPC1.

### Flg22 treatment induces an increase in the size of GAPC1-GFP florescent puncta

During the plant innate immune response, several proteins dynamically re-localize to different sub-cellular compartments [3]. FLS2, the well characterized flagellin receptor, is an example of a protein that is dynamically re-localized after immune activation. FLS2 is localized to the plasma membrane and is intimately connected with the endomembrane system. In the absence of flagellin perception, resting state FLS2 is recycled through the *trans*-Golgi network and early endosomal pathway [50]. When activated, FLS2 is endocytosed, traffics through the endocytic pathway to late endosomes and reaches the multi-vesicular body, presumably for sorting and degradation [50,56]. Thus, flg22 is an excellent probe for cellular re-localization responses during PTI.

**Fig 7:**
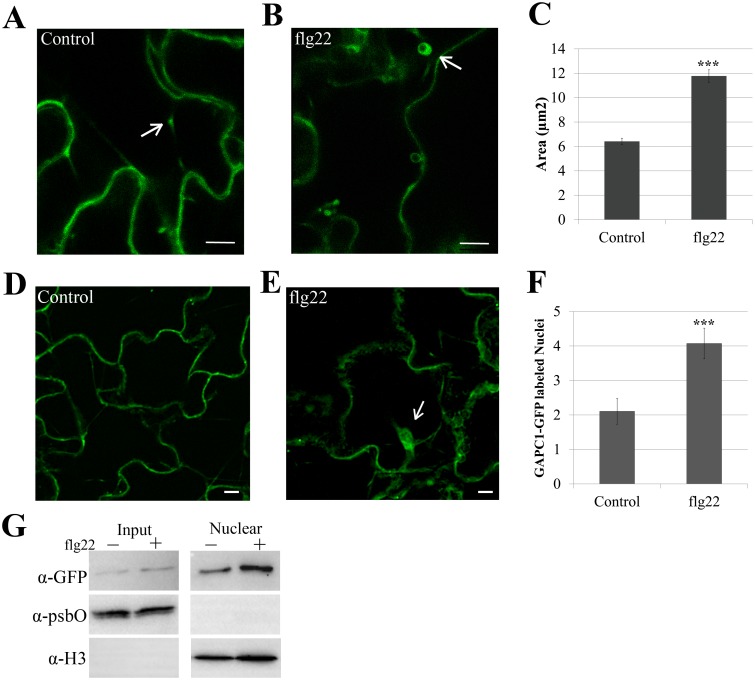
Treatment with flg22 induces an increase in size of GAPC1-GFP labeled vesicles and enhances GAPC1-GFP nuclear localization. Confocal micrographs of the *gapc1* KO complemented with *npro*::*GAPC1-GFP* (line 3–4) show optical sections of leaves from four-week-old plants ±flg22. **(A)** Leaves were infiltrated with a needleless syringe with 10mM MgCl_2_ and imaged after 30 min. Arrows indicate representative vesicles. **(B)** Leaves were infiltrated with 5μM of the elicitor flg22 and imaged after 30 min. Arrows indicate representative vesicles. **(C)** Images were quantified using ImageJ to calculate the area (μM^2^) of GAPC1-GFP labeled vesicles per image across each treatment (n = 10). Statistical differences were detected by a two-tailed Student’s *t* test (α = 0.001). The size of GAPC1-GFP labeled vesicle increases significantly (p< 0.01) at 30 min post-infiltration with flg22. **(D-E)** Nuclear localization of GAPC1-GFP 30 min post-infiltration with 10mM MgCl_2_ **(D)** or 5μM flg22 **(E)**. Arrows indicate individual nuclei. **(F)** Treatment with flg22 enhances GAPC1-GFP nuclear localization. Nuclei were quantified from z-stacks of n = 10 images per treatment. Statistical differences were detected by a two-tailed Student’s *t* test (α = 0.001). **(G)** Nuclear isolation of *npro*::*GAPC1-GFP* seedlings treated 30 min with water or 5μM flg22. Accumulation of GAPC1-GFP in the nuclei of flg22-treated seedlings was detected using α-GFP western blotting. Nuclear enrichment was detected using α-Histone 3 (α-H3) and purity of nuclei was detected using the chloroplast specific photosystem II membrane protein by α-psbO western blotting. Bar = 10μm for all confocal images.

We detected a change in total GAPDH activity during innate immune responses (Fig 4B and 4C) and an association of GAPC1 with diverse compartments in Arabidopsis (Fig 6, [57]). Therefore, GAPC1-GFP localization was examined after elicitation with flg22. Leaves of four-week-old plants were infiltrated with 10mM MgCl_2_ or 5μM flg22 diluted in 10mM MgCl_2_ and imaged 30 min post-infiltration by confocal microscopy. Leaves infiltrated with MgCl_2_ alone exhibited GAPC1-GFP labeled fluorescent puncta that were on average half the size of those in leaves treated with flg22 (Fig 7A, 7B, and 7C). In order to statistically quantify the size change of GAPC1-GFP puncta after flg22 treatment, images from both treatments were pooled and blindly processed for average size. Flg22 treatment was found to induce a significant increase in puncta area (p< 0.01). Arabidopsis GAPC1 and animal GAPDH have also been described as dynamically re-localizing to the nucleus in response to cellular stress [32,58]. Quantification of fluorescently-labeled nuclei from confocal z-stack slices revealed an increase in nuclear-localized GAPC1-GFP after flg22 treatment (Fig 7D, 7E, and 7F). Isolation of nuclei from seedlings treated with water or 5μM flg22 supports enhanced accumulation of GAPC1-GFP in the nucleus after flg22 treatment (Fig 7G). Together, these data indicate a dynamic re-distribution of GAPC1-GFP to the endomembrane system and nucleus during innate immune signaling.

### *GAPA1* and *GAPC1* knockout lines exhibit constitutive autophagy in the absence of nitrogen starvation

**Fig 8:**
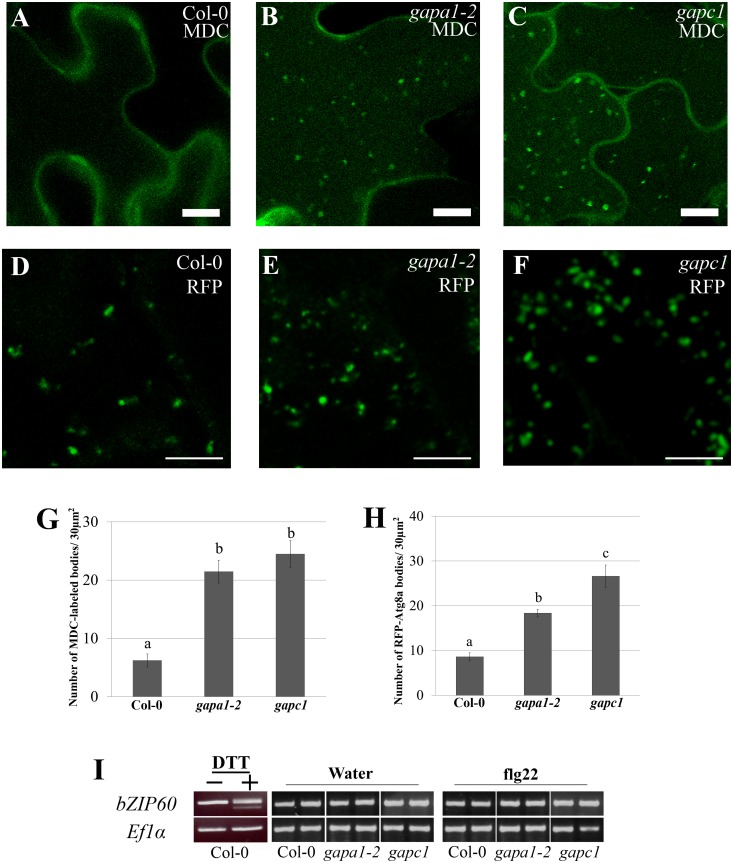
*gapa1-2* and *gapc1* exhibit enhanced basal autophagy. **(A-C)** Confocal micrographs of Col-0, *gapa1-2*, and *gapc1* KO lines show optical sections of leaves from two-week-old plants grown in full MS. Seedlings were assayed with the fluorescent probe MDC. Autophagosomes were stained using 50 μM MDC for 3 h. The *gapa1* and *gapc1* KO lines had many MDC-labeled bodies when grown in full MS, but wild-type Col-0 did not exhibit a significant number of basal MDC-labeled bodies. One μM Concanamycin A or the equivalent amount of solvent DMSO was added 15 h prior to imaging. Bar = 10μm. **(D-F)** Confocal micrographs of 10 day old Col-0, *gapa1-2*, and *gapc1* KO lines transiently transfected with tRFP-Atg8a to visualize cytosolic autophagosomes. Transiently transfected seedlings all exhibit tRFP-Atg8a labeled autophagosomes, with greater numbers present in *gapa1-2* and *gapc1* KOs. Bar = 10 μm. **(G-H)** Images depicted above were quantified using ImageJ to calculate the number of MDC (G) or tRFP-Atg8a (H) labeled bodies in an area of 30 μM^2^. Both *gapa1-2* and *gapc1* seedlings have significant increases in autophagosome number relative to Col-0. Statistical differences were calculated using Fisher’s LSD test (α = 0.05) following a significant F-statistic to compare means of all genotypes. Values represent means ±SE (n = 6). **(I)** The unfolded protein response (UPR) triggered by ER stress is not constitutively activated in *gapa1-2* or *gapc1* KOs. Semi-quantitative RT-PCR analysis of *bZIP60* mRNA demonstrates no cleavage product, indicating that UPR is not activated. The positive control on the left was induced in Col-0 leaves by infiltration with 2 mM DTT. *ELONGATION FACTOR 1α* (*EF1α*) served as a control for equal mRNA levels.

Autophagy is a highly conserved cellular recycling mechanism, involved in degrading unnecessary or damaged materials and organelles during normal growth and development [59]. Although autophagy is an active process in growth and development, few autophagy bodies are present in wild-type plants under normal basal growth conditions [60]. During specific cellular-stresses such as exposure to ROS, endoplasmic reticulum stress or nutrient starvation, autophagy is induced and can lead to programmed cell death [61]. Given that GAPDHs are involved in glucose metabolism and the individual KO lines exhibit enhanced disease resistance, accelerated HR, and enhanced intracellular ROS accumulation, we sought to examine alterations in autophagy responses. In Arabidopsis, autophagy can be induced by nitrogen starvation elicited by growing seedlings on nitrogen-limiting media [60]. For our experiments, two-week-old seedlings were grown first on full strength MS agarose media then transferred to liquid MS or liquid MS lacking nitrogen to induce autophagy for a period of 4–5 days. Monodansylcadaverine (MDC), a fluorescent dye that specifically binds autophagosomes [60,62], was used to visualize autophagy induction by confocal microscopy. Concanamycin A, an inhibitor of vacuolar H+-ATPases, was used to de-acidify the vacuole and allow for visualization of autophagosome accumulation in the vacuole. When grown on full MS media, Col-0 exhibits very few to no autophagy bodies (Fig 8A). Interestingly, *gapa1-2* and *gapc1* seedlings grown on full nutrient media exhibited an enhanced accumulation of autophagosomes in the vacuole as compared to Col-0 (Fig 8B and 8C). Quantification of MDC-labeled puncta within 30 μm^2^ sections revealed a significantly higher number of MDC-labeled autophagosomes in *gapa1-2* and *gapc1* seedlings than Col-0 (Fig 8G). However, there was no gross difference observed between Col-0 and the *gapdh* KO lines in the number of autophagy bodies present under nitrogen starvation (S7 Fig). These data suggest a role for GAPA1 and GAPC1 in the negative regulation of basal autophagy, as the induced autophagy response is phenotypically normal.

To confirm the autophagy phenotype observed with MDC, seedlings were transiently transfected with tRFP-ATG8a and autophagosomes visualized by microscopy. Autophagy is regulated by a set of autophagy-related genes (Atgs) that are highly conserved across eukaryotes [63]. When autophagy is initiated, the ubiquitin-like Atg8 protein can be used as a marker to label cytosolic autophagosomes [60,63]. Autophagosomes labeled with tRFP-Atg8a were present in the cytoplasm of Col-0, *gapa1-2*, and *gapc1* seedlings (Fig 8D, 8E, and 8F). Similar to the MDC-labeling, quantification of the number of fluorescently-labeled puncta in 30 μm^2^ image sections revealed a significantly higher number of autophagosomes present in *gapa1-2* and *gapc1* seedlings compared to Col-0 (Fig 8H).

Autophagy is a diversely regulated process and is intricately connected to cellular glucose metabolism [61]. Not only does glucose availability directly impact glycolysis, but it also impacts glycosylation modifications in the endoplasmic reticulum (ER) [61]. Under nutrient stress a disruption of protein glycosylation in the ER can lead to the unfolded protein response (UPR) and trigger autophagy [61]. To investigate UPR-triggered autophagy, we examined the UPR marker *bZIP60* [64]. During the UPR in plants, IRE1A and IRE1B are activated and cause the cleavage of the mRNA *bZIP60*, which can be visualized by the presence of a doublet after RT-PCR using primers spanning the cleavage site [64,65]. Col-0 treated with 2mM DTT was used as a positive control for induction of the UPR and confirmed the generation of a doublet PCR product (Fig 8I) using previously published primers that span the alternate splicing site [64]. No cleavage of *bZIP60* mRNA was observed in the *gapa1* or *gapc1* KO lines with or without elicitation by flg22 (Fig 8I). Without cleavage of *bZIP60* mRNA, canonical UPR activation should not be responsible for the induction of autophagy.

## Discussion

In this manuscript, we genetically investigated the importance of five of the seven phosphorylating GAPDHs, revealing enhanced defense responses in individual KO lines. GAPDHs are differentially regulated by a variety of post-translational modifications, some of which have been linked to transcriptional reprogramming and endosomal trafficking in animals [12,18]. Our data provide evidence that plant GAPDHs serve roles outside of glycolysis and the Calvin cycle. The sub-cellular localization of GAPC1-GFP fluorescent puncta and their subsequent change in size after flg22 treatment indicates a response consistent with either ROS-induced protein aggregation or fusion of GAPC1-GFP associated endosomal compartments [66,67]. Additionally, we see a change in GAPDH glycolytic activity during immune signaling. Impacts on plant immunity imparted by individual isoforms are likely influenced by complex genetic regulation.

Our analyses of *GAPDH* KO lines revealed that individual KOs affected immune responses. Individual KO lines exhibited enhanced disease resistance to virulent and avirulent *Pst* DC3000, accelerated cell death in response to avirulent *Pst* DC3000, enhanced basal expression of the defense marker gene *PR1*, and enhanced intracellular ROS accumulation. Furthermore, the *gapc1* and *gapa1* possessed an increased basal autophagy phenotype. These effects may be linked as downstream consequences of the heightened basal ROS detected in each KO. Exogenous ROS application has been shown to enhance *PR1* gene expression, autophagy flux, and protein aggregation [66–70]. Although individual *GAPDH* KO lines exhibited similar phenotypic effects, their magnitude varied. qRT-PCR analyses revealed expression of individual *GAPDH* isoforms were regulated in a complex manner across single KO lines. In most instances, there was a compounding effect where mutation of a single *GAPDH* resulted in the down-regulation of multiple *GAPDH* isoforms. For example, *GAPA1* and *GAPA2* were significantly down-regulated across all single KO lines, mimicking transcriptional responses observed during PTI (Figs 3B, 3C, and 4C). During PTI in wild-type plants, *GAPC1* mRNA was significantly up-regulated four-fold compared to controls 3h post-flg22 treatment, while transcription of photosynthetic *GAPDHs* was down-regulated at this time point. *GAPDH* is frequently used as a control to normalize gene expression during qPCR. Our data highlight that *GAPDH* expression dynamically changes upon flg22 perception. Therefore, *GAPDH* expression should be interrogated before use or alternative marker genes should be used to normalize gene expression [71].

Total glycolytic GAPDH enzymatic activity increased during infection with virulent or avirulent *Pst* DC3000 as well as after perception of flg22. Enhanced glycolytic activity has been linked to promotion of cell survival [14,72]. Additionally, a primary output of glycolysis is pyruvate, a direct scavenger of cellular ROS [73]. Thus, in wild-type plants undergoing immune responses, enhancing glycolysis may be important for providing elevated levels of the ROS scavenger pyruvate. Furthermore, plant defense signaling is an energetic process, highlighted by the well-known tradeoff between growth and defense [74]. Glycolysis generates ATP. Thus, an increase in glycolytic GAPDH activity during pathogen perception could provide additional energy required for global cellular reprogramming towards defense. In mammals, GAPDH is required for glycolytic ATP-driven rapid vesicular transport [75]. We also observed GAPC1-GFP associating with vesicles (Figs 6 and 7), which could provide energy enabling vesicular movement.

Using the ROS sensitive probe H_2_DCFDA, we found all tested *GAPDH* KO lines exhibited enhanced intracellular ROS accumulation. GAPA1, GAPA2, and GAPB catalyze the reductive step in the Calvin cycle with concomitant oxidation of NADPH to NADP+ [76]. Interestingly, *GAPA1* and *GAPA2* mRNA expression levels were significantly lower in all KO lines. NADP+ is important as an electron acceptor for protons accumulating at the thylakoid membrane generated during photosynthesis. A reduction of NADP+ can lead to an increase in ROS production at the thylakoid membrane due to excess proton accumulation [24]. In addition to Calvin-cycle mediated regulation of intracellular ROS production, GAPDH proteins are also sensitive to regulation by ROS themselves. Our results in combination with previous experiments demonstrated that hydrogen peroxide treatment inactivated GAPA1, GAPC1, GAPC2, and GAPCp1/2 enzymatic activity *in vitro* [27–29]. Large scale proteomic studies have identified S-Nitrosylation of Arabidopsis GAPDHs during infection with avirulent *Pst* DC3000 and in response to treatment with nitric oxide [25,26,77]. It will be important to determine if the oxidative state of GAPDH is monitored and if GAPDHs directly contribute to ROS quenching in plants.

Depending on the pathogen and recognized effector, autophagy can promote cell death or survival [16]. Autophagy is required to limit the spread of ETI induced cell death after infection of *Nicotiana benthamiana* with Tobacco Mosaic Virus [78]. A pro-death role for autophagy has also been described in the case of ETI triggered by *Pst* DC3000 *AvrRps4* in *Arabidopsis* Ws-0 [79]. Although autophagy was required for wild-type HR triggered by AvrRps4, it was not required for normal HR responses to *Pst* DC3000 effectors AvrRpt2 or AvrRpm1 [79]. Therefore, the accelerated HR we observed in response to *Pst* DC3000 AvrRpt2 infiltration is likely due to enhanced defense priming in the *GAPDH* KO lines. ROS have also been shown to be essential for the formation of autophagosomes in mammalian cells and Arabidopsis [69,70]. We see enhanced ROS and *PR1* expression in the *GAPDH* KOs, indicating autophagy may be induced as a pro-survival mechanism against accumulating ROS.

Previous studies have demonstrated primarily cytoplasmic localization for GAPC1, with occasional nuclear accumulation [32,73,80]. Our *npro*::*GAPC1-GFP* complemented lines also exhibited a similar localization pattern. Overexpression of GAPC1 in protoplasts enables enhanced nuclear accumulation as well as association with mitochondria and the actin cytoskeleton [32,81]. However, these subcellular localizations were not detected in stable native promoter GAPC1-YFP lines [32]. In animal cells as well as plant roots, GAPDH can dynamically re-localize to the nucleus upon oxidative or cold stress [20,32,80]. We also detected enhanced nuclear localization and a significant alteration in the size of GAPC1-GFP fluorescent puncta during PTI. In its monomeric form, human nuclear GAPDH is active as a uracil-DNA-glycosylase [82]. Plant chloroplast isoform GAPB was demonstrated to have nuclear uracil-DNA-glycosylase activity [57,83]. This moonlighting GAPDH activity may be an important component of monitoring DNA damage.

We also detected GAPC1-GFP as plasma membrane associated and in small mobile puncta. We demonstrate that GAPC1-GFP is sensitive to the PI3K inhibitor Wortmannin which is a chemical inhibitor of autophagy. Recently, GAPC1 and GAPC2 were reported to bind membrane-localized Phospholipase D (PLD) and its downstream product Phosphatidic Acid [30,84]. Furthermore, the oxidized form of GAPCs significantly enhanced PLD enzymatic activity [30]. The association of GAPC1 with PLD under oxidizing conditions could account for an increase in the size of vesicles associated with GAPC1-GFP after perception of flg22 (Fig 7B).

Here, we provide evidence that GAPDHs can influence plant immune responses and GAPC1 exhibits diverse and dynamic cellular localization upon flagellin perception. Phenotypes observed in the KOs such as the accumulation of reactive oxygen species and induction of basal autophagy support GAPDH mediated regulation of metabolic checkpoints. Future research investigating the role of nuclear GAPC1 will determine if plant GAPDHs, like their animal counterparts, are involved in transcriptional reprogramming during times of cellular stress. We have provided evidence supporting GAPDHs as pro-survival molecules in plants, negatively regulating cell death in response to pathogen challenge. Due to a lack of gross morphological phenotypes in single *gapdh* KOs, individual members may be promising targets for genome editing to enhance crop disease resistance.

## Materials and Methods

### Plant materials and growth conditions

T-DNA insertion lines for *GAPA1* (SALK_138567 and SALK_145802; *gapa1-1* and *gapa1-2* respectively), *GAPC1* (SALK_010839), and *GAPC2* (SALK_016935) were obtained from the SALK institute, genotyped, and homozygous KO lines were verified by RT-PCR. Homozygous seed for *GAPCp1* (SAIL_390_G10 and SALK_052938; *gapCp1-1* and *gapCp1-2* respectively) and *GAPCp2* (SALK_137288, SALK_008979; *gapCp2-1* and *gapCp2-2* respectively) were previously described [34]. Plants were grown in a controlled environmental chamber at 23°C with a 10-h light/14-h dark photoperiod under a light intensity of 85 μE/m^2^/s. For all the experiments, 4–5 week old plants were used.

Transgenic *npro*::*GAPC1-GFP* lines were generated using the floral dip method [85], in order to complement the *gapc1* KO. The length of native promoter we used was 811bp, and it was PCR amplified and cloned as an in-frame fusion to genomic *GAPC1* in pENTR (Invitrogen). Next, the *GAPC1* construct was transferred into the binary vector pGWB4 using gateway technology to generate a C-terminal GFP tag [86]. Transgenic plants were selected on 50μg/mL hygromycin. T3 homozygous lines were used for all experiments. All PCR primers used for genotyping and cloning are listed in S1 Table.

### Bacterial strains and inoculations

*Pst* DC3000 and *Pst* DC3000 (AvrRpt2), were grown on nutrient yeast-glycerol (NYG) plates for 30 h, then cultured at 28°C in NYG media for 48 h. *Pst* DC3000 (AvrRpt2) expressed AvrRpt2 from the broad-host range vector pDSK519 [87]. Antibiotics were used for plate selection at the following concentrations: 25 μg/ml kanamycin, 100 μg/ml rifampicin, and 35 μg/ml chloramphenicol. For dip inoculation, *Arabidopsis* plants were grown in a mesh covered pot to facilitate submergence for 30 sec of the aerial portion into bacterial suspension containing 1×10^9^ CFU/ml bacteria in 10 mM MgCl_2_with 0.02% silwet L-77. Inoculated plants were left covered with a plastic dome for 3h. At 4 days post inoculation, leaves were surface sterilized for 30 sec in 70% ethanol and bacterial populations were determined as described by Kim and colleagues [88]. All experiments were repeated at least three times, with a minimum of six biological replicates per time point.

### HR and electrolyte leakage

For both HR and electrolyte leakage, *Arabidopsis* Col-0 and *GAPDH* KO leaves were infiltrated using a needleless syringe with 4×10^7^ CFU/ml of *Pst* DC3000 and *Pst* DC3000 (AvrRpt2). After infiltration, plants were placed under a light bank (100 μE/m^2^/s) and HR was scored at 10 h post inoculation. For electrolyte leakage, two leaves per plant were infiltrated across four biological replicates per genotype. Total tissue was harvested using a cork borer to generate 1.5 cm^2^ of leaf discs (six total leaf discs). Leaf discs were placed in distilled water (20mL in a 50mL conical tube) for 1h and individual biological replicates kept separate. Leaf discs were transferred to a 12-well tissue culture plate (Corning) containing 4mL of distilled water per well and placed under the light bank. Conductivity was measured using the Orion 3 Star conductivity meter (Thermo Scientific). All experiments were repeated at least three times.

### Phylogenetic analysis

MEGA6 was used to perform phylogenetic analysis on GAPDH amino acid sequences and draw the un-rooted tree [37]. A maximum-likelihood tree construction was used based on the JTT matrix-based model with bootstraps.

### RT-PCR and qRT-PCR analysis

Total RNA was extracted using the TRI zol Reagent (Invitrogen) according to manufacturer’s instructions, and subsequently incubated with RNase-free DNase I (Invitrogen) to remove genomic DNA contamination. RNA was extracted from three biological replicates per treatment. Each biological sample comprised two leaves from a single plant, and the pooled 2μg of RNA was used as a template for reverse transcription with Promega M-MLV reverse transcriptase in the presence of 0.5μg/μl oligo(dT) primers. Equal amounts of first-strand cDNAs were used as templates for RT-PCR amplification using the primers listed in supplemental S1 Table. Semi-quantitative RT-PCR was run for 30 cycles for *gapa1-2* lines and 35 cycles for *gapCp* lines. Quantitative real-time PCR reactions used Bio-Rad SsoFast EvaGreen Supermix according to manufacturer’s directions using a CFX96 Touch (Bio-Rad). Thermocyling parameters began with a first step at 95°C for 30 sec and 39 cycles afterwards alternating between 5 sec at 95°C and 15 sec at 60°C. A melting curve followed the final cycle and ran 5s at 65°C and 5 s at 95°C. Gene expression was normalized against the *Arabidopsis ELONGATION FACTOR 1-ALPHA* (At5g60390).

### Protoplast preparation

Protoplasts were prepared enzymatically according to previously described methods [86]. After isolation, protoplasts were re-suspended in W1 solution (0.5 M mannitol, 4 mM MES, pH 5.7, 20 mM KCl) after the rinse steps with W5 (154 mM NaCl, 125 mM CaCl_2_, 5 mM KCl, 2 mM MES, pH 5.7) and allowed to sit and recover for 5h before treatment. After resting, protoplasts were quantified with a hemacytometer and aliquoted into a 24 well Corning Costar cell culture plate where they were diluted with W1 to 1 x 10^5^ cells/ 200μL. The cell culture plate was moved to a light bank where the protoplasts were left under bright light for 1h. Prior to imaging, 750nM 2',7'-dichlorodihydrofluorescein diacetate (H_2_DCFDA, CalBioChem) was added and cells incubated in the dark for 12–15 min. Images used for quantification of *gapa1* and Col-0 (Fig 5) were obtained using a Leica DM 5000B epifluorescent microscope with a GFP cube (excitation 470/40, emission 525/50). All other genotypes were imaged using the Axio Imager M2 microscope (Zeiss, Germany) using a 40X objective (EC Plan-NEOFLUAR 40X/ 0.75, Zeiss). Fluorescence was quantified using ImageJ from a minimum of 25 protoplasts across 10 or more images per genotype.

### Western blot analysis

SDS-PAGE and subsequent immunoblotting were performed according to standard procedures [89]. GAPC1-GFP immunoblots were performed with Anti-GFP (ab290, Abcam) rabbit polyclonal antibody at a dilution of 1:8,000. Anti-cF6BP immunoblots were performed using rabbit polyclonal antibody at a dilution of 1: 5,000 (Agrisera). Anti-RIN4 immunoblotting used affinity purified antisera from rabbit at 1:3,000. Rabbit polyclonal antibodies anti-Histone 3 (ab1791, Abcam) and anti-psbO (ab65563, Abcam) were used at 1:1,000 and 1:3,000, respectively. Secondary goat anti-rabbit IgG-HRP conjugate (Biorad) was used at a dilution of 1:3,000 for detection via enhanced chemiluminescence (Pierce). GAPDH immunoblots were performed using anti-GAPDH (GenScript) goat polyclonal antibody at a dilution of 1:500. Secondary bovine anti-goat IgG-HRP (Santa Cruz Biotechnology) was used at a dilution of 1:3,000 for detection via enhanced chemiluminescence (Pierce). Plasmids containing the seven phosphorylating GAPDH cDNAs in a pET28a backbone were transfected into *E*. *coli*. Recombinant protein was purified on Ni-NTA Agarose beads (Qiagen) and used for Western blotting.

### GAPDH enzymatic assay

Leaves from 4–5 week old rosette leaves were ground in liquid nitrogen by mortar and pestle. Each sample contained pooled leaves from 3–4 plants. Homogenates were otherwise prepared as described [22]. To the frozen, ground tissue, 600 μl of buffer containing 50mM Tris-HCl, pH 8.0, 5mM EDTA, 1mM phenylmethylsulfonyl fluoride, and 2mM 2-mercaptoethanol was added. The homogenate was transferred to an eppendorf tube and centrifuged at 12,000 g for 20 min at 4°C. The supernatant was collected and protein content quantified by Pierce 660 protein assay (Thermo Scientific) and normalized across all samples and 30μg of total leaf protein was used for all activity assays. Cytosolic GAPDH activity was assayed spectrophotometrically using a Spectramax Plus384 spectrophotometer (Molecular Devices) at 340nm by the reduction of NAD+, as described [22]. Photosynthetic GAPDH activity was also assayed as described previously with minor modifications [76]. 3-phosphoglycerate kinase was omitted in the photosynthetic GAPDH activity assay since it is present and abundant in the leaf extract [90]. Experiments were repeated a minimum of three times, and data was normalized to the control and all runs combined for statistical analysis. In the recombinant GAPDH activity assays, 1.5 μg of total protein was used. Hydrogen peroxide at specified concentrations was added just prior to the initiation of the assay.

### Confocal microscopy

All confocal microscopy was performed using a Zeiss LSM710 confocal microscope equipped with a LDC-apochromat 40×/1.1W Korr M27 water-immersion objective (NA 1.1). GFP was excited at 488nm, emission collected at 500–550nm for all experiments except MDC treatments. After treatment with 50 μM MDC, GFP emission was collected at 510–560nm. Leaves incubated with 1 μM FM464 (Invitrogen) were excited at 488nm and emission collected at 620–660nm. MDC was visualized using UV laser and emission of 467–510nm.

For flg22 treatment, leaves of four-week-old plants were infiltrated using a needleless syringe with 5μM flg22 (GenScript, 80.1% purity) or water and imaged after 30 min. Images were randomized and aggregate size was blindly quantified using ImageJ. Brefeldin A (BFA, Sigma) bodies and endosomal networks were imaged using three-week-old seedlings submerged in 1μM FM4-64 for 3h with or without addition of 30μM BFA 1.5h prior to imaging.

Autophagy was examined according to [60], with slight modifications. Seeds were sown on full MS agarose plates and grown for 10 days in 16h light/ 8h dark at 23°C. Seedlings were then transferred to liquid media containing MS or nitrogen-free MS (*Phyto*Technology Laboratories) for 4–5 more days under the same growth conditions. The night before imaging, 1μM Concanamycin A (Sigma) or equivalent volume of DMSO was added to each well. Three hours before imaging, 50μM MDC was added and plates were wrapped in foil and kept in the dark.

Transient expression of 2x35S::tRFP-Atg8a was as previously described [91], with some modifications. After a four day co-cultivation period with Agrobacterium GV3101 the co-cultivation media was removed and seedlings were washed three times with distilled water. Seedlings were then re-suspended in full MS media and imaged by confocal microscopy. All image analysis was performed using a combination of software tools, Zen 2012 software (Carl Zeiss), ImageJ (http://rsbweb.nih.gov/ij/) and Image Pro Plus (Media Cybernetics, Rockville, MD).

### Isolation of nuclei

Isolation of Arabidopsis nuclei was carried out as previously described with some modifications [92]. Two week old seedlings were transferred to a six-well culture plate and treated ± 5 μM flg22 for 30 min. One gram of seedlings from each treatment was frozen in liquid nitrogen for nuclear isolation. Seedlings were ground in liquid nitrogen and re-suspended in 5mL Extraction Buffer (2 M hexylene glycol, 20 mM PIPES-KOH (pH 7.0), 10 mM MgCl_2_, 1 mM Spermidine, 1 mM Spermine, 1 mM 2-Mercaptoethanol, 1% Triton X-100). The suspension was stirred at 4°C for 10 minutes and then filtered through two layers of cheesecloth stacked with two layers of Miracloth (EMD Millipore).

Percoll suspensions of 30% and 80% Percoll were prepared in Gradient Buffer (0.5M hexylene glycol, 5 mM PIPES-KOH (pH 7.0), 10mM MgCl_2_, 1 mM 2-Mercaptoethanol, 1% Triton X-100). Three mL of 30% Percoll were added to a 15 mL conical tube and under-laid with 3 mL of 80% Percoll. The plant extract was pipetted on top and the sample was centrifuged at 1000g for 30 minutes at 4°C. Nuclei were extracted from the interface between the 30% and 80% Percoll layers into a 2 mL tube. The nuclear fraction was brought to 0.5 mL volume with Gradient buffer and under-laid with 30% Percoll and centrifuged at 2000g for 10 minutes at 4°C. After the supernatent was completely removed and the pellet was re-suspended in 0.5 mL gradient buffer, 0.5 mL of 30% Percoll was again under-laid and the 2000g centrifugation repeated. The pellet was then re-suspended in Laemmli buffer, quantified for equal loading using Pierce 660 protein assay (Thermo Scientific), and diluted to 1 μg/ 10 μl for SDS-PAGE gel analysis.

## Supporting Information

S1 Fig Characterization of *gapa1*, *gapCp1* and *gapCp2* knockout lines. **(A)** Diagram of *GAPA1* illustrating locations of two independent T-DNA insertion sites. Primers used for RT-PCR were QRT-A1-F/R. **(B)** RT-PCR of *GAPA1* T-DNA insertion lines *gapa1-1* (SALK_138567) and *gapa1-2* (SALK_145802). *GAPA1* is not expressed in the T-DNA insertion lines. Actin2 was used as a reference. **(C-D)** Diagrams of *GAPCp1* (Top, At1g79530) and *GAPCp2* (Bottom, At1g16300), respectively, illustrating locations of the T-DNA insertion sites. Primers used for RT-PCR were QRT-Cp1-F/R and QRT-Cp2-F/R. **(E)** RT-PCR of *GAPCp1* and *GAPCp2* on T-DNA insertion lines *gapCp1-1* (SAIL_390_G10), *gapCp1-2* (SALK_052938), *gapCp2-1* (SALK_137288) and *gapCp2-2* (SALK_008979). *GAPCp1* is not expressed in *gapCp1-1* or *gapCp1-2*, and *GAPCp2* is not expressed in *gapCp2-1* or *gapCp2-2*. ELONGATION FACTOR 1-α (At5g60390) was used as a reference.
(TIF)

S2 Fig Detection of endogenous GAPDH in wild-type Col-0 and *GAPC1-GFP* complemented lines using α-GAPDH. **(A)** Antibody specificity of α-GAPDH against recombinant GAPDH protein purified from *E*. *coli*. Top: The GAPDH antibody detects GAPC1, GAPC2, GAPCp1 and GAPCp2. A weak band can be seen for GAPB (single asterisk). Neither GAPA1 nor GAPA2 are detected. Bottom: Coomassie stained gel demonstrating relative protein abundance and purity within each sample. Single asterisks in GAPA1, GAPA2 and GAPB lanes mark correct band size for those proteins. 0.25 μg of protein was loaded for western blotting and 0.35 μg for Coomassie staining. **(B)** Col-0, *gapc1*, and T3 complementation lines transformed with *npro*::*GAPC1-GFP* were subjected to western blotting using α-GAPDH. Two independent transformation lines are shown: 3–4 and 9–6. Using α-GAPDH western blotting, the GAPC1-GFP band is detected around 65kD and is indicated by a single asterisk. Endogenous GAPDH is indicated by a double asterisk at 40kD, and is present in all samples. **(C)** The lines described in (B) were subjected to α-GFP western blotting, revealing a 65kD band for GAPC1-GFP.
(TIF)

S3 Fig Growth curves on *GAPDH* KO lines four days post dip inoculation with *Pst* DC3000 *AvrRpt2* at a concentration of (1x10^9^ CFU mL^-1^). All *GAPDH* KOs exhibit reduced bacterial growth. Values represent means ±SE, n = 4. Statistical differences were detected by a two-tailed Student’s *t* test (p<0.05 *, p<0.01 **, p<0.001 ***) compared to the Col-0 control. Experiment was repeated a minimum of 3 times with similar results.
(TIF)

S4 Fig GAPDH protein levels do not grossly change during PTI or ETI. **(A) Leaves from four-week-old Col-0 plants were infiltrated with 5μM flg22 or water and samples taken at the indicated time points.** Eight micrograms of leaf protein were subjected to western blotting with α-GAPDH. **(B)** Leaves from four-week-old Col-0 plants were infiltrated with 10mM MgCl_2_ or a bacterial suspension containing *Pst* DC3000 *AvrRpt2* or empty vector (EV) at a concentration of 4×10^7^ CFU mL^-1^. Samples were harvested 8 h post-infiltration. Eight micrograms of leaf protein were subjected to western blotting with α-GAPDH.
(TIF)

S5 Fig ROS phenotypes of *GAPDH* knockouts and recombinant proteins. **(A)** Analyses of the flg22-induced ROS burst in Col-0 and *GAPDH* KO lines. Leaf discs were taken from four-week-old plants and floated in water for 24h prior to treatment with 100nM flg22. ROS was detected as fluorescence using a luminol-based assay. Relative light units (RLUs) were quantified using a Berthold luminometer and maximum RLU values were used for quantification. Values represent means ± SE (n≥112) of ≥ 9 combined runs. Statistical differences were detected by a two-tailed Student’s *t* test (α = 0.01) compared to wild-type Col-0. **(B)** Recombinant GAPDH proteins are inhibited in a dose-dependent manner by H_2_O_2_. Recombinant proteins purified from *E*. *coli* were used in a glycolytic GAPDH activity assay with or without the addition of H_2_O_2_. Error bars indicate standard deviation on two separate runs with n = 3 for each.
(TIF)

S6 Fig GAPDH is localized to the nuclear, cytosolic and microsomal fractions. Nuclear, cytosolic and microsomal fractions from two-week-old seedlings were isolated using the Minute Plasma Membrane Protein Isolation kit (Invent biotechnologies, Inc). Western blotting using α-GAPDH demonstrates primarily cytosolic localization with some protein found in the microsomal fraction and less in the nuclear fraction. Western blotting with marker proteins was used to verify enrichment of individual fractions. α-F6BP is a cytosolic marker and α-RIN4 is a microsomal marker. A total of 8μg of protein was loaded per lane.
(TIF)

S7 Fig Induced autophagy in *gapa1-2* and *gapc1* is indistinguishable from wild-type Col-0. Two-week-old seedlings were grown on MS media without nitrogen for four days, and then incubated with the 50μM of the fluorescent dye MDC for 3 h. Autophagy bodies were visualized by confocal microscopy. Two independent images are shown for each genotype. Scale bar = 10 μm.
(TIF)

S1 Table Primers used in experiments. (5’ to 3’)
(PDF)

## References

[1] Ronald PC, Beutler B (2010) Plant and animal sensors of conserved microbial signatures. Science 330: 1061–1064. 10.1126/science.1189468 21097929

[2] Spoel SH, Dong X (2012) How do plants achieve immunity? Defence without specialized immune cells. Nat Rev Immunol 12: 89–100. 10.1038/nri3141 22273771

[3] Henry E, Yadeta KA, Coaker G (2013) Recognition of bacterial plant pathogens: local, systemic and transgenerational immunity. New Phytol 199: 908–915. 10.1111/nph.12214 23909802 PMC3740753

[4] Zipfel C (2009) Early molecular events in PAMP-triggered immunity. Curr Opin Plant Biol 12: 414–420. 10.1016/j.pbi.2009.06.003 19608450

[5] Thomma BPHJ, Nürnberger T, Joosten MHAJ (2011) Of PAMPs and Effectors: The Blurred PTI-ETI Dichotomy. The Plant Cell 23: 4–15. 10.1105/tpc.110.082602 21278123 PMC3051239

[6] Mackey D, Belkhadir Y, Alonso JM, Ecker JR, Dangl JL (2003) Arabidopsis RIN4 Is a Target of the Type III Virulence Effector AvrRpt2 and Modulates RPS2-Mediated Resistance. Cell 112: 379–389. 12581527 10.1016/s0092-8674(03)00040-0

[7] Bomblies K, Lempe J, Epple P, Warthmann N, Lanz C, et al (2007) Autoimmune Response as a Mechanism for a Dobzhansky-Muller-Type Incompatibility Syndrome in Plants. PLoS Biol 5: e236 17803357 10.1371/journal.pbio.0050236 PMC1964774

[8] Takahashi A, Casais C, Ichimura K, Shirasu K (2003) HSP90 interacts with RAR1 and SGT1 and is essential for RPS2-mediated disease resistance in Arabidopsis. Proc Natl Acad Sci U S A 100: 11777–11782. 14504384 10.1073/pnas.2033934100 PMC208834

[9] Peart JR, Lu R, Sadanandom A, Malcuit I, Moffett P, et al (2002) Ubiquitin ligase-associated protein SGT1 is required for host and nonhost disease resistance in plants. Proc Natl Acad Sci U S A 99: 10865–10869. 12119413 10.1073/pnas.152330599 PMC125064

[10] Wang RY-L, Nagy PD (2008) Tomato bushy stunt virus Co-Opts the RNA-Binding Function of a Host Metabolic Enzyme for Viral Genomic RNA Synthesis. Cell Host & Microbe 3: 178–187. 10.1016/j.chom.2008.02.005 18329617

[11] Nicaise V, Joe A, Jeong Br, Korneli C, Boutrot F, et al (2013) Pseudomonas HopU1 modulates plant immune receptor levels by blocking the interaction of their mRNAs with GRP7. EMBO J. 32: 701–712. 10.1038/emboj.2013.15 23395902 PMC3590987

[12] Tristan C, Shahani N, Sedlak TW, Sawa A (2011) The diverse functions of GAPDH: views from different subcellular compartments. Cell Signal 23: 317–323. 10.1016/j.cellsig.2010.08.003 20727968 PMC3084531

[13] Colell A, Green DR, Ricci JE (2009) Novel roles for GAPDH in cell death and carcinogenesis. Cell Death Differ 16: 1573–1581. 10.1038/cdd.2009.137 19779498

[14] Colell A, Ricci JE, Tait S, Milasta S, Maurer U, et al (2007) GAPDH and autophagy preserve survival after apoptotic cytochrome c release in the absence of caspase activation. Cell 129: 983–997. 17540177 10.1016/j.cell.2007.03.045

[15] Gao X, Wang X, Pham TH, Feuerbacher LA, Lubos ML, et al (2013) NleB, a bacterial effector with glycosyltransferase activity, targets GAPDH function to inhibit NF-kappaB activation. Cell Host & Microbe 13: 87–99. 10.1016/j.chom.2012.11.010 23332158 PMC3553500

[16] Green DR, Galluzzi L, Kroemer G (2014) Cell biology. Metabolic control of cell death. Science 345: 1250256 10.1126/science.1250256 25237106 PMC4219413

[17] Cheng SC, Quintin J, Cramer RA, Shepardson KM, Saeed S, et al (2014) mTOR- and HIF-1alpha-mediated aerobic glycolysis as metabolic basis for trained immunity. Science 345: 1250684 10.1126/science.1250684 25258083 PMC4226238

[18] Zaffagnini M, Fermani S, Costa A, Lemaire SD, Trost P (2013) Plant cytoplasmic GAPDH: redox post-translational modifications and moonlighting properties. Front Plant Sci 4: 450 10.3389/fpls.2013.00450 24282406 PMC3824636

[19] Sirover MA (2011) On the functional diversity of glyceraldehyde-3-phosphate dehydrogenase: biochemical mechanisms and regulatory control. Biochim Biophys Acta 1810: 741–751. 10.1016/j.bbagen.2011.05.010 21640161

[20] Sirover MA (2012) Subcellular dynamics of multifunctional protein regulation: mechanisms of GAPDH intracellular translocation. J Cell Biochem 113: 2193–2200. 10.1002/jcb.24113 22388977 PMC3350569

[21] Petersen J, Brinkmann H, Cerff R (2003) Origin, evolution, and metabolic role of a novel glycolytic GAPDH enzyme recruited by land plant plastids. J Mol Evol 57: 16–26. 12962302 10.1007/s00239-002-2441-y

[22] Rius SP, Casati P, Iglesias AA, Gomez-Casati DF (2008) Characterization of Arabidopsis lines deficient in GAPC-1, a cytosolic NAD-dependent glyceraldehyde-3-phosphate dehydrogenase. Plant Physiol 148: 1655–1667. 10.1104/pp.108.128769 18820081 PMC2577239

[23] Baalmann E, Backhausen JE, Rak C, Vetter S, Scheibe R (1995) Reductive modification and nonreductive activation of purified spinach chloroplast NADP-dependent glyceraldehyde-3-phosphate dehydrogenase. Arch Biochem Biophys 324: 201–208. 8554310 10.1006/abbi.1995.0031

[24] Price GD, Evans J, Caemmerer S, Yu J-W, Badger M (1995) Specific reduction of chloroplast glyceraldehyde-3-phosphate dehydrogenase activity by antisense RNA reduces CO2 assimilation via a reduction in ribulose bisphosphate regeneration in transgenic tobacco plants. Planta 195: 369–378. 7766043 10.1007/BF00202594

[25] Maldonado-Alconada A, Echevarría-Zomeño S, Lindermayr C, Redondo-López I, Durner J, et al (2011) Proteomic analysis of Arabidopsis protein S-nitrosylation in response to inoculation with Pseudomonas syringae. Acta Physiologiae Plantarum 33: 1493–1514.

[26] Romero-Puertas MC, Campostrini N, Matte A, Righetti PG, Perazzolli M, et al (2008) Proteomic analysis of S-nitrosylated proteins in Arabidopsis thaliana undergoing hypersensitive response. Proteomics 8: 1459–1469. 10.1002/pmic.200700536 18297659

[27] Zaffagnini M, Michelet L, Marchand C, Sparla F, Decottignies P, et al (2007) The thioredoxin-independent isoform of chloroplastic glyceraldehyde-3-phosphate dehydrogenase is selectively regulated by glutathionylation. FEBS J 274: 212–226. 17140414 10.1111/j.1742-4658.2006.05577.x

[28] Holtgrefe S, Gohlke J, Starmann J, Druce S, Klocke S, et al (2008) Regulation of plant cytosolic glyceraldehyde 3-phosphate dehydrogenase isoforms by thiol modifications. Physiol Plant 133: 211–228. 10.1111/j.1399-3054.2008.01066.x 18298409

[29] Hancock JT, Henson D, Nyirenda M, Desikan R, Harrison J, et al (2005) Proteomic identification of glyceraldehyde 3-phosphate dehydrogenase as an inhibitory target of hydrogen peroxide in Arabidopsis. Plant Physiol Biochem 43: 828–835. 16289945 10.1016/j.plaphy.2005.07.012

[30] Guo L, Devaiah SP, Narasimhan R, Pan X, Zhang Y, et al (2012) Cytosolic glyceraldehyde-3-phosphate dehydrogenases interact with phospholipase Ddelta to transduce hydrogen peroxide signals in the Arabidopsis response to stress. Plant Cell 24: 2200–2212. 10.1105/tpc.111.094946 22589465 PMC3442596

[31] Baek D, Jin Y, Jeong JC, Lee HJ, Moon H, et al (2008) Suppression of reactive oxygen species by glyceraldehyde-3-phosphate dehydrogenase. Phytochemistry 69: 333–338. 17854848 10.1016/j.phytochem.2007.07.027

[32] Vescovi M, Zaffagnini M, Festa M, Trost P, Lo Schiavo F, et al (2013) Nuclear accumulation of cytosolic glyceraldehyde-3-phosphate dehydrogenase in cadmium-stressed Arabidopsis roots. Plant Physiol 162: 333–346. 10.1104/pp.113.215194 23569110 PMC3641213

[33] Preston GM (2000) Pseudomonas syringae pv. tomato: the right pathogen, of the right plant, at the right time. Mol Plant Pathol 1: 263–275. 10.1046/j.1364-3703.2000.00036.x 20572973

[34] Munoz-Bertomeu J, Cascales-Minana B, Mulet JM, Baroja-Fernandez E, Pozueta-Romero J, et al (2009) Plastidial glyceraldehyde-3-phosphate dehydrogenase deficiency leads to altered root development and affects the sugar and amino acid balance in Arabidopsis. Plant Physiol 151: 541–558. 10.1104/pp.109.143701 19675149 PMC2754643

[35] Chisholm ST, Coaker G, Day B, Staskawicz BJ (2006) Host-microbe interactions: shaping the evolution of the plant immune response. Cell 124: 803–814. 16497589 10.1016/j.cell.2006.02.008

[36] Durrant WE, Dong X (2004) Systemic acquired resistance. Annu Rev Phytopathol 42: 185–209. 15283665 10.1146/annurev.phyto.42.040803.140421

[37] Tamura K, Stecher G, Peterson D, Filipski A, Kumar S (2013) MEGA6: Molecular Evolutionary Genetics Analysis version 6.0. Mol Biol Evol 30: 2725–2729. 10.1093/molbev/mst197 24132122 PMC3840312

[38] Rius SP, Casati P, Iglesias AA, Gomez-Casati DF (2006) Characterization of an Arabidopsis thaliana mutant lacking a cytosolic non-phosphorylating glyceraldehyde-3-phosphate dehydrogenase. Plant Mol Biol 61: 945–957. 16927206 10.1007/s11103-006-0060-5

[39] Straus MR, Rietz S, Ver Loren van Themaat E, Bartsch M, Parker JE (2010) Salicylic acid antagonism of EDS1-driven cell death is important for immune and oxidative stress responses in Arabidopsis. Plant J 62: 628–640. 10.1111/j.1365-313X.2010.04178.x 20163553

[40] Shapiguzov A, Vainonen JP, Wrzaczek M, Kangasjarvi J (2012) ROS-talk—how the apoplast, the chloroplast, and the nucleus get the message through. Front Plant Sci 3: 292 10.3389/fpls.2012.00292 23293644 PMC3530830

[41] Colussi C, Albertini MC, Coppola S, Rovidati S, Galli F, et al (2000) H2O2-induced block of glycolysis as an active ADP-ribosylation reaction protecting cells from apoptosis. FASEB J 14: 2266–2276. 11053248 10.1096/fj.00-0074com

[42] Gómez-Gómez L, Boller T (2000) FLS2: An LRR Receptor—like Kinase Involved in the Perception of the Bacterial Elicitor Flagellin in Arabidopsis. Molecular Cell 5: 1003–1011. 10911994 10.1016/s1097-2765(00)80265-8

[43] Steiner L (1989) Antibodies—a Laboratory Manual—Harlow,E, Lane,D. Nature 341: 32–32.

[44] Torres MA, Dangl JL, Jones JD (2002) Arabidopsis gp91phox homologues AtrbohD and AtrbohF are required for accumulation of reactive oxygen intermediates in the plant defense response. Proc Natl Acad Sci U S A 99: 517–522. 11756663 10.1073/pnas.012452499 PMC117592

[45] Chandra-Shekara AC, Gupte M, Navarre D, Raina S, Raina R, et al (2006) Light-dependent hypersensitive response and resistance signaling against Turnip Crinkle Virus in Arabidopsis. Plant J 45: 320–334. 16412080 10.1111/j.1365-313X.2005.02618.x

[46] Foyer CH, Noctor G (2005) Redox Homeostasis and Antioxidant Signaling: A Metabolic Interface between Stress Perception and Physiological Responses. The Plant Cell Online 17: 1866–1875. 10.1105/tpc.105.033589 PMC1167537 15987996

[47] Bryksin AV, Laktionov PP (2008) Role of glyceraldehyde-3-phosphate dehydrogenase in vesicular transport from golgi apparatus to endoplasmic reticulum. Biochemistry (Mosc) 73: 619–625. 18620527 10.1134/s0006297908060011

[48] Glaser PE, Gross RW (1995) Rapid plasmenylethanolamine-selective fusion of membrane bilayers catalyzed by an isoform of glyceraldehyde-3-phosphate dehydrogenase: discrimination between glycolytic and fusogenic roles of individual isoforms. Biochemistry 34: 12193–12203. 7547960 10.1021/bi00038a013

[49] Tisdale EJ (2001) Glyceraldehyde-3-phosphate dehydrogenase is required for vesicular transport in the early secretory pathway. J Biol Chem 276: 2480–2486. 11035021 10.1074/jbc.M007567200

[50] Beck M, Zhou J, Faulkner C, MacLean D, Robatzek S (2012) Spatio-temporal cellular dynamics of the Arabidopsis flagellin receptor reveal activation status-dependent endosomal sorting. Plant Cell 24: 4205–4219. 10.1105/tpc.112.100263 23085733 PMC3516521

[51] Emans N, Zimmermann S, Fischer R (2002) Uptake of a fluorescent marker in plant cells is sensitive to brefeldin A and wortmannin. Plant Cell 14: 71–86. 11826300 10.1105/tpc.010339 PMC150552

[52] Nebenführ A, Ritzenthaler C, Robinson DG (2002) Brefeldin A: Deciphering an Enigmatic Inhibitor of Secretion. Plant Physiology 130: 1102–1108. 12427977 10.1104/pp.011569 PMC1540261

[53] Bolte S, Talbot C, Boutte Y, Catrice O, Read ND, et al (2004) FM-dyes as experimental probes for dissecting vesicle trafficking in living plant cells. J Microsc 214: 159–173. 15102063 10.1111/j.0022-2720.2004.01348.x

[54] Bonazzi M, Spano S, Turacchio G, Cericola C, Valente C, et al (2005) CtBP3/BARS drives membrane fission in dynamin-independent transport pathways. Nat Cell Biol 7: 570–580. 15880102 10.1038/ncb1260

[55] De Matteis MA, Di Girolamo M, Colanzi A, Pallas M, Di Tullio G, et al (1994) Stimulation of endogenous ADP-ribosylation by brefeldin A. Proc Natl Acad Sci U S A 91: 1114–1118. 8302839 10.1073/pnas.91.3.1114 PMC521464

[56] Spallek T, Beck M, Ben Khaled S, Salomon S, Bourdais G, et al (2013) ESCRT-I mediates FLS2 endosomal sorting and plant immunity. PLoS Genet 9: e1004035 10.1371/journal.pgen.1004035 24385929 PMC3873229

[57] Anderson LE, Ringenberg MR, Carol AA (2004) Cytosolic glyceraldehyde-3-P dehydrogenase and the B subunit of the chloroplast enzyme are present in the pea leaf nucleus. Protoplasma 223: 33–43. 15004741 10.1007/s00709-003-0030-6

[58] Hara MR, Agrawal N, Kim SF, Cascio MB, Fujimuro M, et al (2005) S-nitrosylated GAPDH initiates apoptotic cell death by nuclear translocation following Siah1 binding. Nat Cell Biol 7: 665–674. 15951807 10.1038/ncb1268

[59] Li F, Vierstra RD (2012) Autophagy: a multifaceted intracellular system for bulk and selective recycling. Trends Plant Sci 17: 526–537. 10.1016/j.tplants.2012.05.006 22694835

[60] Woo J, Park E, Dinesh-Kumar SP (2014) Differential processing of Arabidopsis ubiquitin-like Atg8 autophagy proteins by Atg4 cysteine proteases. Proc Natl Acad Sci U S A 111: 863–868. 10.1073/pnas.1318207111 24379391 PMC3896200

[61] Dodson M, Darley-Usmar V, Zhang J (2013) Cellular metabolic and autophagic pathways: traffic control by redox signaling. Free Radic Biol Med 63: 207–221. 10.1016/j.freeradbiomed.2013.05.014 23702245 PMC3729625

[62] Contento AL, Xiong Y, Bassham DC (2005) Visualization of autophagy in Arabidopsis using the fluorescent dye monodansylcadaverine and a GFP-AtATG8e fusion protein. The Plant Journal 42: 598–608. 15860017 10.1111/j.1365-313X.2005.02396.x

[63] Mizushima N, Yoshimori T, Ohsumi Y (2011) The role of Atg proteins in autophagosome formation. Annu Rev Cell Dev Biol 27: 107–132. 10.1146/annurev-cellbio-092910-154005 21801009

[64] Moreno AA, Mukhtar MS, Blanco F, Boatwright JL, Moreno I, et al (2012) IRE1/bZIP60-mediated unfolded protein response plays distinct roles in plant immunity and abiotic stress responses. PLoS One 7: e31944 10.1371/journal.pone.0031944 22359644 PMC3281089

[65] Deng Y, Humbert S, Liu JX, Srivastava R, Rothstein SJ, et al (2011) Heat induces the splicing by IRE1 of a mRNA encoding a transcription factor involved in the unfolded protein response in Arabidopsis. Proc Natl Acad Sci U S A 108: 7247–7252. 10.1073/pnas.1102117108 21482766 PMC3084119

[66] Nakajima H, Amano W, Fujita A, Fukuhara A, Azuma Y-T, et al (2007) The Active Site Cysteine of the Proapoptotic Protein Glyceraldehyde-3-phosphate Dehydrogenase Is Essential in Oxidative Stress-induced Aggregation and Cell Death. Journal of Biological Chemistry 282: 26562–26574. 17613523 10.1074/jbc.M704199200

[67] Nakajima H, Amano W, Kubo T, Fukuhara A, Ihara H, et al (2009) Glyceraldehyde-3-phosphate Dehydrogenase Aggregate Formation Participates in Oxidative Stress-induced Cell Death. Journal of Biological Chemistry 284: 34331–34341. 10.1074/jbc.M109.027698 19837666 PMC2797201

[68] Chen Z, Silva H, Klessig DF (1993) Active oxygen species in the induction of plant systemic acquired resistance by salicylic acid. Science 262: 1883–1886. 8266079 10.1126/science.8266079

[69] Scherz-Shouval R, Shvets E, Fass E, Shorer H, Gil L, et al (2007) Reactive oxygen species are essential for autophagy and specifically regulate the activity of Atg4. EMBO J 26: 1749–1760. 17347651 10.1038/sj.emboj.7601623 PMC1847657

[70] Xiong Y, Contento AL, Nguyen PQ, Bassham DC (2007) Degradation of oxidized proteins by autophagy during oxidative stress in Arabidopsis. Plant Physiol 143: 291–299. 17098847 10.1104/pp.106.092106 PMC1761971

[71] Czechowski T, Stitt M, Altmann T, Udvardi MK, Scheible WR (2005) Genome-wide identification and testing of superior reference genes for transcript normalization in Arabidopsis. Plant Physiol 139: 5–17. 16166256 10.1104/pp.105.063743 PMC1203353

[72] Krawczyk CM, Holowka T, Sun J, Blagih J, Amiel E, et al (2010) Toll-like receptor-induced changes in glycolytic metabolism regulate dendritic cell activation. Blood 115: 4742–4749. 10.1182/blood-2009-10-249540 20351312 PMC2890190

[73] Giandomenico AR, Cerniglia GE, Biaglow JE, Stevens CW, Koch CJ (1997) The importance of sodium pyruvate in assessing damage produced by hydrogen peroxide. Free Radic Biol Med 23: 426–434. 9214579 10.1016/s0891-5849(97)00113-5

[74] Belkhadir Y, Yang L, Hetzel J, Dangl JL, Chory J (2014) The growth-defense pivot: crisis management in plants mediated by LRR-RK surface receptors. Trends Biochem Sci 39: 447–456. 10.1016/j.tibs.2014.06.006 25089011 PMC4177940

[75] Zala D, Hinckelmann MV, Yu H, Lyra da Cunha MM, Liot G, et al (2013) Vesicular glycolysis provides on-board energy for fast axonal transport. Cell 152: 479–491. 10.1016/j.cell.2012.12.029 23374344

[76] Sparla F, Pupillo P, Trost P (2002) The C-terminal extension of glyceraldehyde-3-phosphate dehydrogenase subunit B acts as an autoinhibitory domain regulated by thioredoxins and nicotinamide adenine dinucleotide. J Biol Chem 277: 44946–44952. 12270927 10.1074/jbc.M206873200

[77] Lindermayr C, Saalbach G, Durner J (2005) Proteomic identification of S-nitrosylated proteins in Arabidopsis. Plant Physiol 137: 921–930. 15734904 10.1104/pp.104.058719 PMC1065393

[78] Liu Y, Schiff M, Czymmek K, Talloczy Z, Levine B, et al (2005) Autophagy regulates programmed cell death during the plant innate immune response. Cell 121: 567–577. 15907470 10.1016/j.cell.2005.03.007

[79] Hofius D, Schultz-Larsen T, Joensen J, Tsitsigiannis DI, Petersen NH, et al (2009) Autophagic components contribute to hypersensitive cell death in Arabidopsis. Cell 137: 773–783. 10.1016/j.cell.2009.02.036 19450522

[80] Bae MS, Cho EJ, Choi EY, Park OK (2003) Analysis of the Arabidopsis nuclear proteome and its response to cold stress. Plant J 36: 652–663. 14617066 10.1046/j.1365-313x.2003.01907.x

[81] Wojtera-Kwiczor J, Gross F, Leffers HM, Kang M, Schneider M, et al (2012) Transfer of a Redox-Signal through the Cytosol by Redox-Dependent Microcompartmentation of Glycolytic Enzymes at Mitochondria and Actin Cytoskeleton. Front Plant Sci 3: 284 10.3389/fpls.2012.00284 23316205 PMC3540817

[82] Meyer-Siegler K, Mauro DJ, Seal G, Wurzer J, deRiel JK, et al (1991) A human nuclear uracil DNA glycosylase is the 37-kDa subunit of glyceraldehyde-3-phosphate dehydrogenase. Proc Natl Acad Sci U S A 88: 8460–8464. 1924305 10.1073/pnas.88.19.8460 PMC52528

[83] Wang X, Sirover MA, Anderson LE (1999) Pea chloroplast glyceraldehyde-3-phosphate dehydrogenase has uracil glycosylase activity. Arch Biochem Biophys 367: 348–353. 10395754 10.1006/abbi.1999.1261

[84] Kim SC, Guo L, Wang X (2013) Phosphatidic acid binds to cytosolic glyceraldehyde-3-phosphate dehydrogenase and promotes its cleavage in Arabidopsis. J Biol Chem 288: 11834–11844. 10.1074/jbc.M112.427229 23504314 PMC3636871

[85] Clough SJ, Bent AF (1998) Floral dip: a simplified method for Agrobacterium-mediated transformation of Arabidopsis thaliana. Plant J 16: 735–743. 10069079 10.1046/j.1365-313x.1998.00343.x

[86] Nakagawa T, Kurose T, Hino T, Tanaka K, Kawamukai M, et al (2007) Development of series of gateway binary vectors, pGWBs, for realizing efficient construction of fusion genes for plant transformation. J Biosci Bioeng 104: 34–41. 17697981 10.1263/jbb.104.34

[87] Mudgett MB, Staskawicz BJ (1999) Characterization of the Pseudomonas syringae pv. tomato AvrRpt2 protein: demonstration of secretion and processing during bacterial pathogenesis. Mol Microbiol 32: 927–941. 10361296 10.1046/j.1365-2958.1999.01403.x

[88] Kim MG, da Cunha L, McFall AJ, Belkhadir Y, DebRoy S, et al (2005) Two Pseudomonas syringae type III effectors inhibit RIN4-regulated basal defense in Arabidopsis. Cell 121: 749–759. 15935761 10.1016/j.cell.2005.03.025

[89] Harlow E, Lane DP (1988) Antibodies: A Laboratory Manual. Cold Spring Harbor (New York): Cold Spring Harbor Press.

[90] Edelman M, Hallick RB, Chua NH (1982) Methods in chloroplast molecular biology. Amsterdam; New York New York, N.Y.: Elsevier Biomedical Press; Sole distributors for the U.S.A. and Canada, Elsevier Science Pub. Co. xiii, 1140 p. p.

[91] Li JF, Park E, von Arnim AG, Nebenfuhr A (2009) The FAST technique: a simplified Agrobacterium-based transformation method for transient gene expression analysis in seedlings of Arabidopsis and other plant species. Plant Methods 5: 6 10.1186/1746-4811-5-6 19457242 PMC2693113

[92] Folta KM, Kaufman LS (2006) Isolation of Arabidopsis nuclei and measurement of gene transcription rates using nuclear run-on assays. Nat Protoc 1: 3094–3100. 17406505 10.1038/nprot.2006.471

