# Supplementary figures and images for: Beyond Glycolysis: GAPDHs Are Multi-functional Enzymes Involved in Regulation of ROS, Autophagy, and Plant Immune Responses
Source: PLoS Genet. 2015 Apr 28;11(4):e1005199. doi: 10.1371/journal.pgen.1005199 (PMC4412566; doi:10.1371/journal.pgen.1005199)

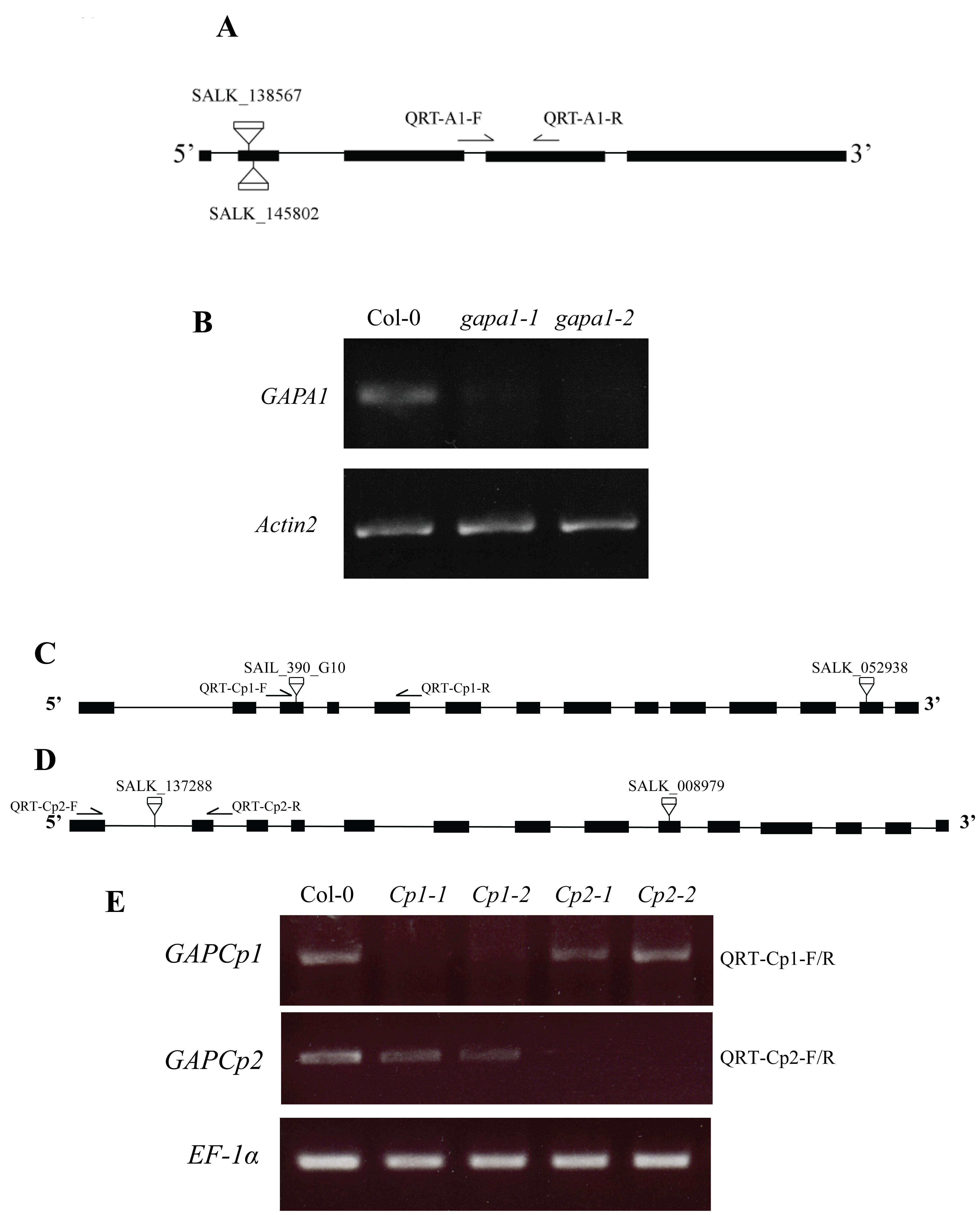

Supplement: S1 Fig — (A) Diagram of GAPA1 illustrating locations of two independent T-DNA insertion sites. Primers used for RT-PCR were QRT-A1-F/R. (B) RT-PCR of GAPA1 T-DNA insertion lines gapa1-1 (SALK_138567) and gapa1-2 (SALK_145802). GAPA1 is not expressed in the T-DNA insertion lines. Actin2 was used as a reference. (C-D) Diagrams of GAPCp1 (Top, At1g79530) and GAPCp2 (Bottom, At1g16300), respectively, illustrating locations of the T-DNA insertion sites. Primers used for RT-PCR were QRT-Cp1-F/R and QRT-Cp2-F/R. (E) RT-PCR of GAPCp1 and GAPCp2 on T-DNA insertion lines gapCp1-1 (SAIL_390_G10), gapCp1-2 (SALK_052938), gapCp2-1 (SALK_137288) and gapCp2-2 (SALK_008979). GAPCp1 is not expressed in gapCp1-1 or gapCp1-2, and GAPCp2 is not expressed in gapCp2-1 or gapCp2-2. ELONGATION FACTOR 1-α (At5g60390) was used as a reference. (TIF) [file pgen.1005199.s001.tif]

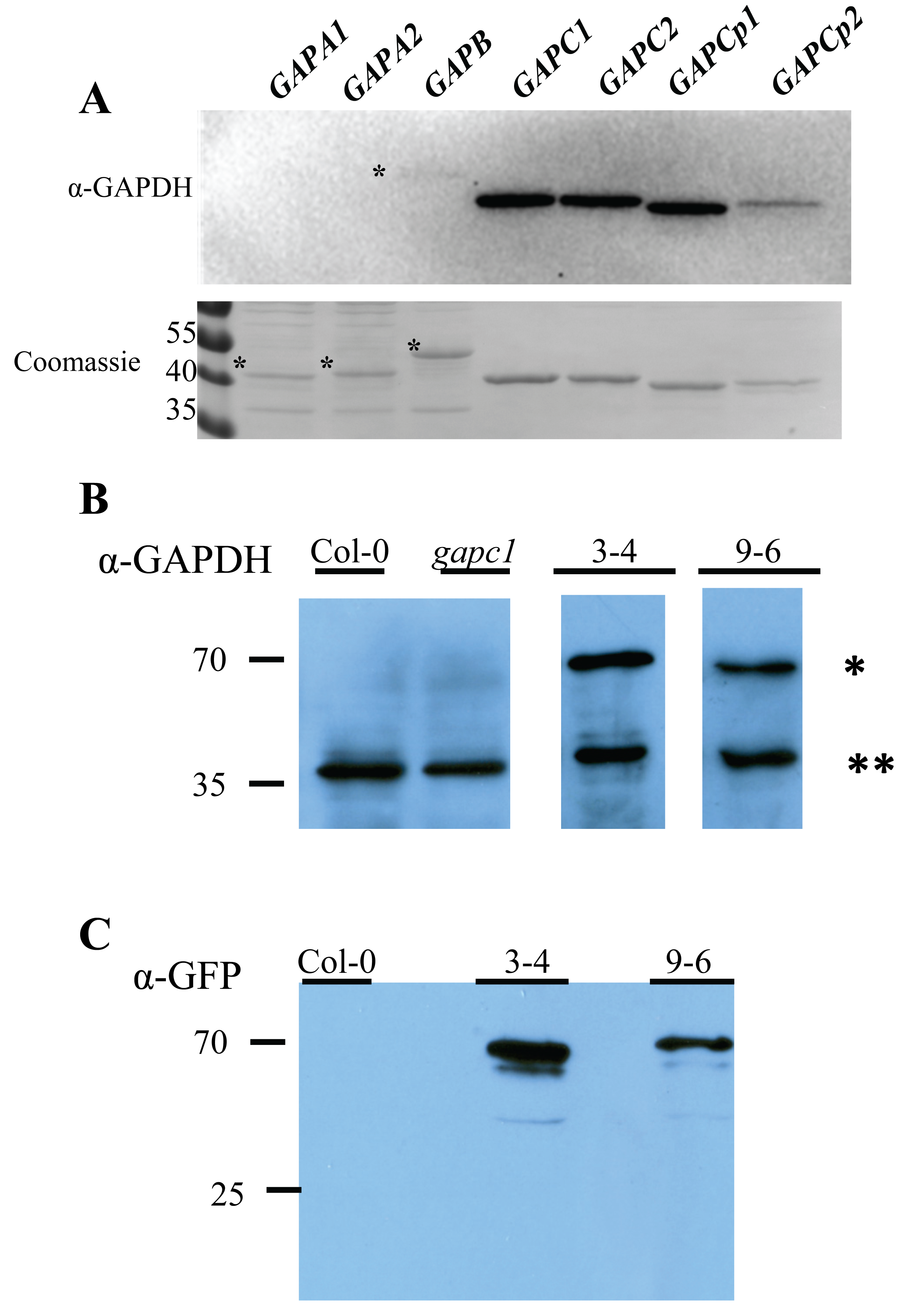

Supplement: S2 Fig — (A) Antibody specificity of α-GAPDH against recombinant GAPDH protein purified from E. coli. Top: The GAPDH antibody detects GAPC1, GAPC2, GAPCp1 and GAPCp2. A weak band can be seen for GAPB (single asterisk). Neither GAPA1 nor GAPA2 are detected. Bottom: Coomassie stained gel demonstrating relative protein abundance and purity within each sample. Single asterisks in GAPA1, GAPA2 and GAPB lanes mark correct band size for those proteins. 0.25 μg of protein was loaded for western blotting and 0.35 μg for Coomassie staining. (B) Col-0, gapc1, and T3 complementation lines transformed with npro::GAPC1-GFP were subjected to western blotting using α-GAPDH. Two independent transformation lines are shown: 3–4 and 9–6. Using α-GAPDH western blotting, the GAPC1-GFP band is detected around 65kD and is indicated by a single asterisk. Endogenous GAPDH is indicated by a double asterisk at 40kD, and is present in all samples. (C) The lines described in (B) were subjected to α-GFP western blotting, revealing a 65kD band for GAPC1-GFP. (TIF) [file pgen.1005199.s002.tif]

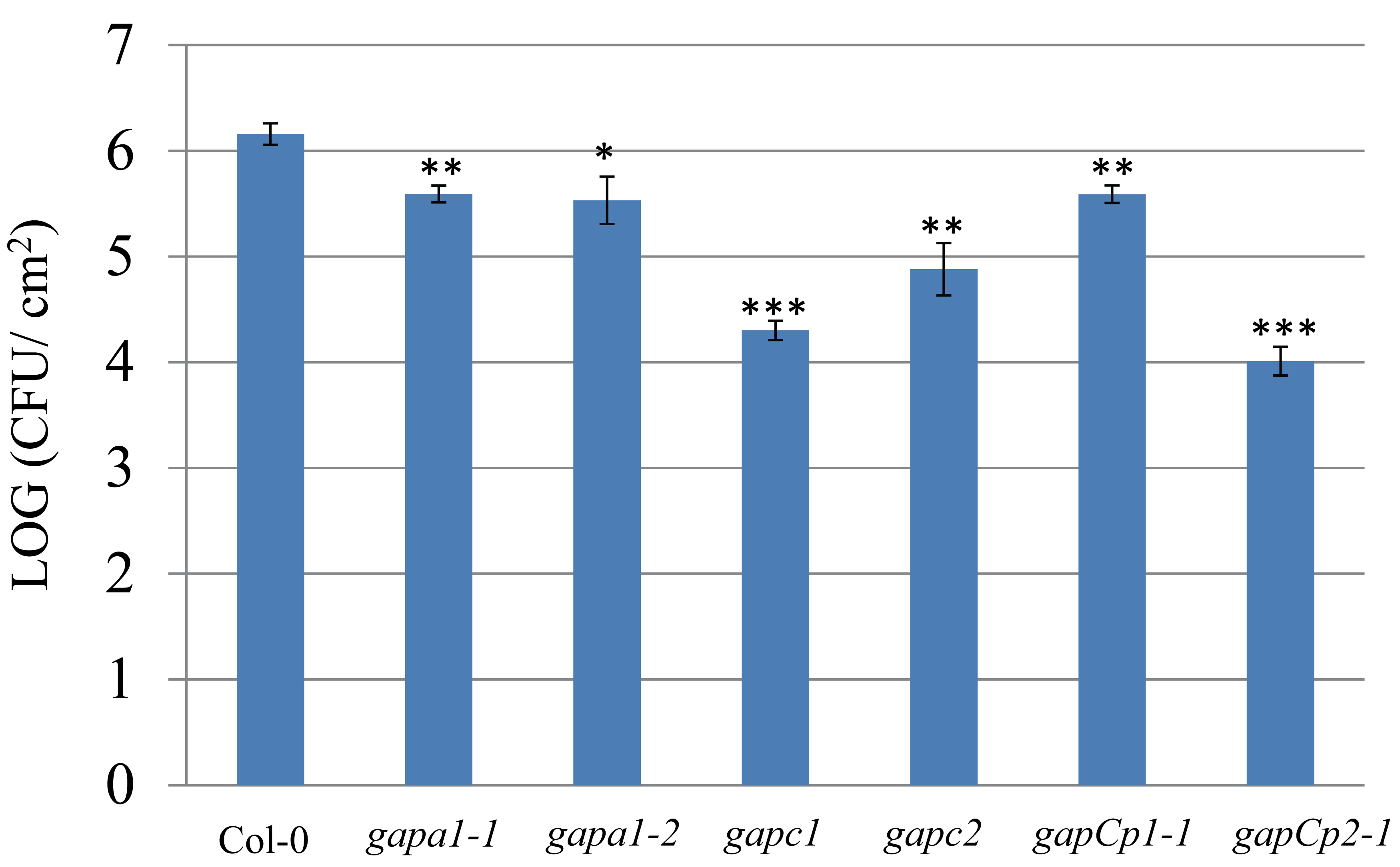

Supplement: S3 Fig — All GAPDH KOs exhibit reduced bacterial growth. Values represent means ±SE, n = 4. Statistical differences were detected by a two-tailed Student’s t test (p<0.05 *, p<0.01 **, p<0.001 ***) compared to the Col-0 control. Experiment was repeated a minimum of 3 times with similar results. (TIF) [file pgen.1005199.s003.tif]

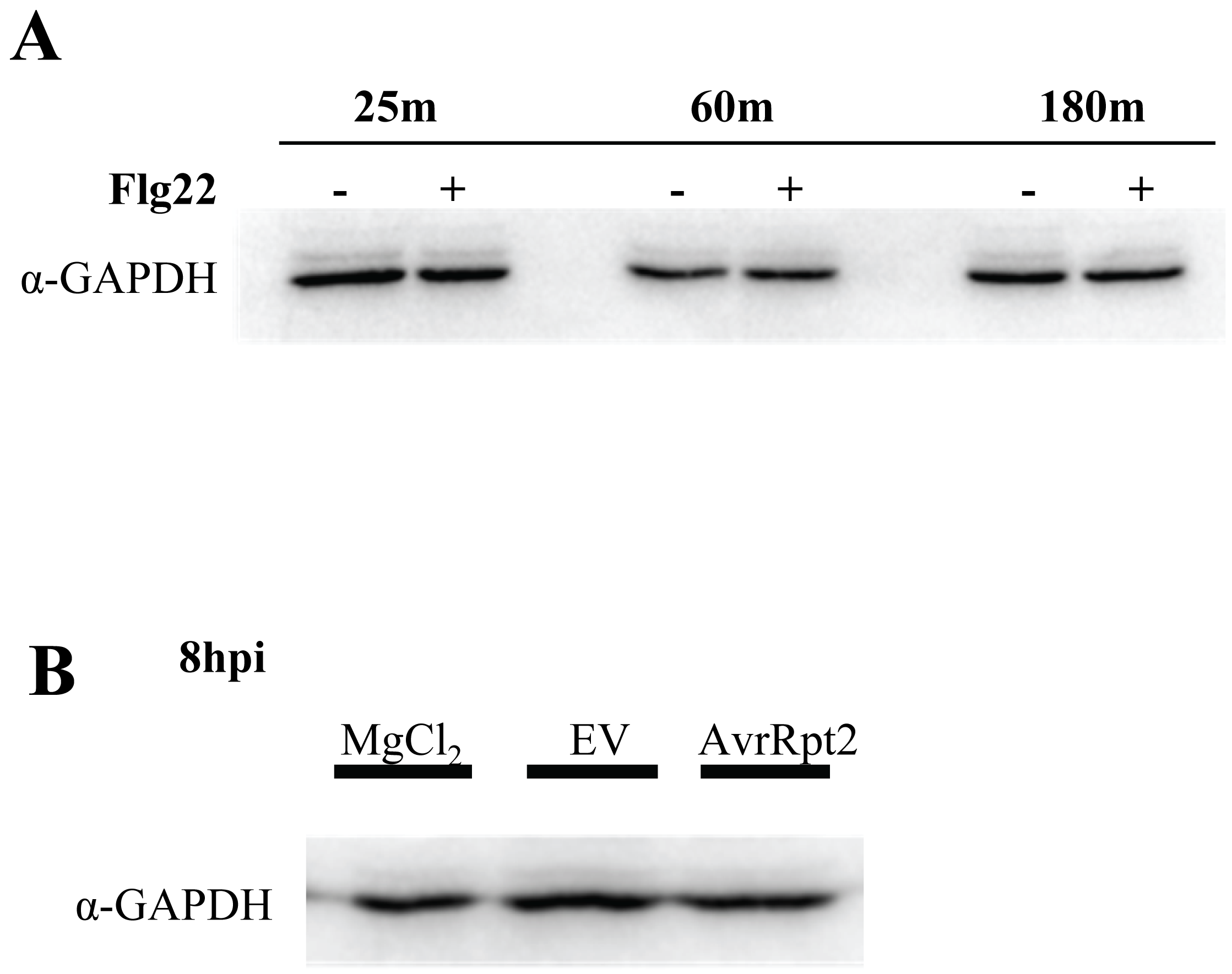

Supplement: S4 Fig — (A) Leaves from four-week-old Col-0 plants were infiltrated with 5μM flg22 or water and samples taken at the indicated time points. Eight micrograms of leaf protein were subjected to western blotting with α-GAPDH. (B) Leaves from four-week-old Col-0 plants were infiltrated with 10mM MgCl2 or a bacterial suspension containing Pst DC3000 AvrRpt2 or empty vector (EV) at a concentration of 4×10^7^ CFU mL-1. Samples were harvested 8 h post-infiltration. Eight micrograms of leaf protein were subjected to western blotting with α-GAPDH. (TIF) [file pgen.1005199.s004.tif]

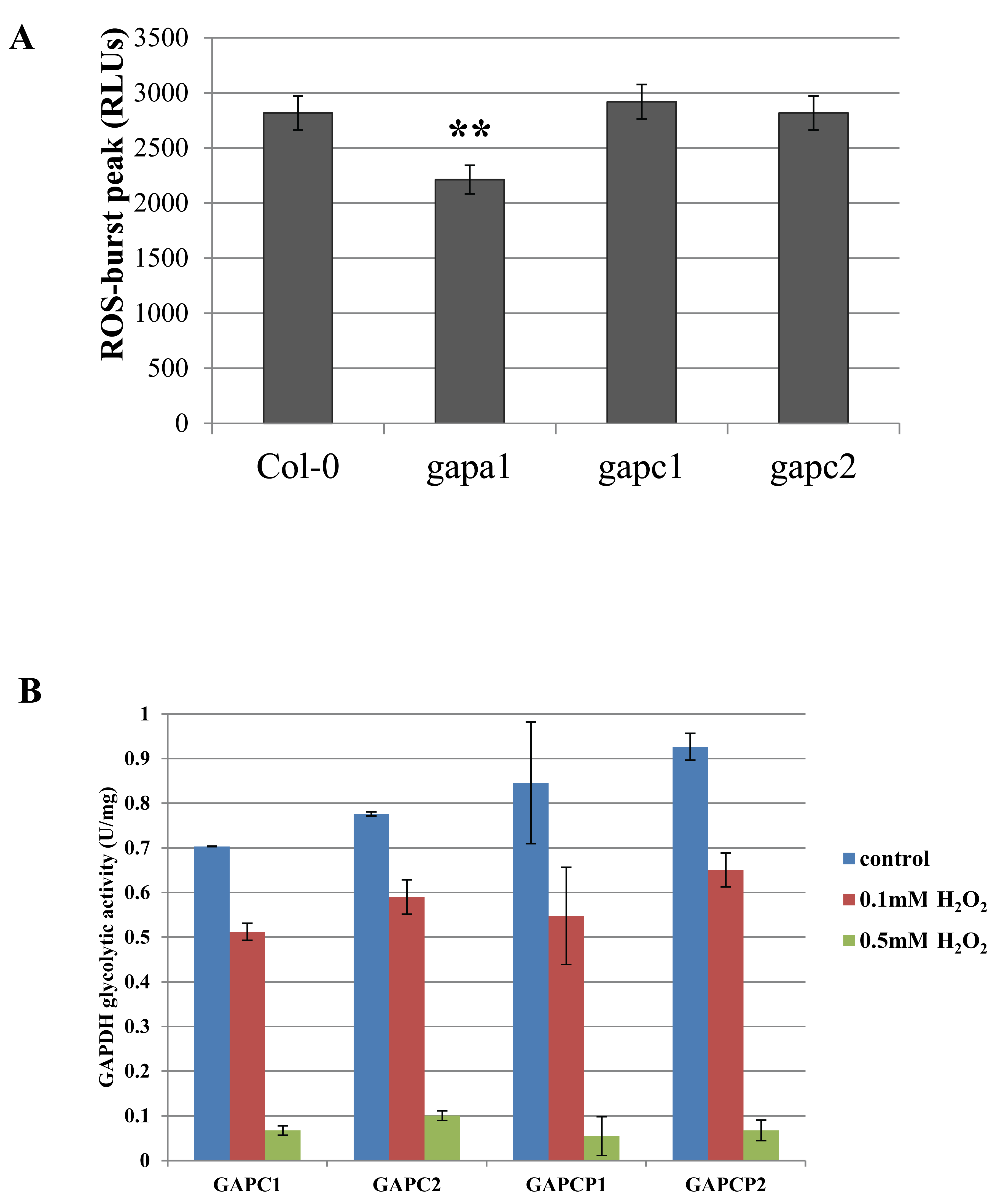

Supplement: S5 Fig — (A) Analyses of the flg22-induced ROS burst in Col-0 and GAPDH KO lines. Leaf discs were taken from four-week-old plants and floated in water for 24h prior to treatment with 100nM flg22. ROS was detected as fluorescence using a luminol-based assay. Relative light units (RLUs) were quantified using a Berthold luminometer and maximum RLU values were used for quantification. Values represent means ± SE (n≥112) of ≥ 9 combined runs. Statistical differences were detected by a two-tailed Student’s t test (α = 0.01) compared to wild-type Col-0. (B) Recombinant GAPDH proteins are inhibited in a dose-dependent manner by H2O2. Recombinant proteins purified from E. coli were used in a glycolytic GAPDH activity assay with or without the addition of H2O2. Error bars indicate standard deviation on two separate runs with n = 3 for each. (TIF) [file pgen.1005199.s005.tif]

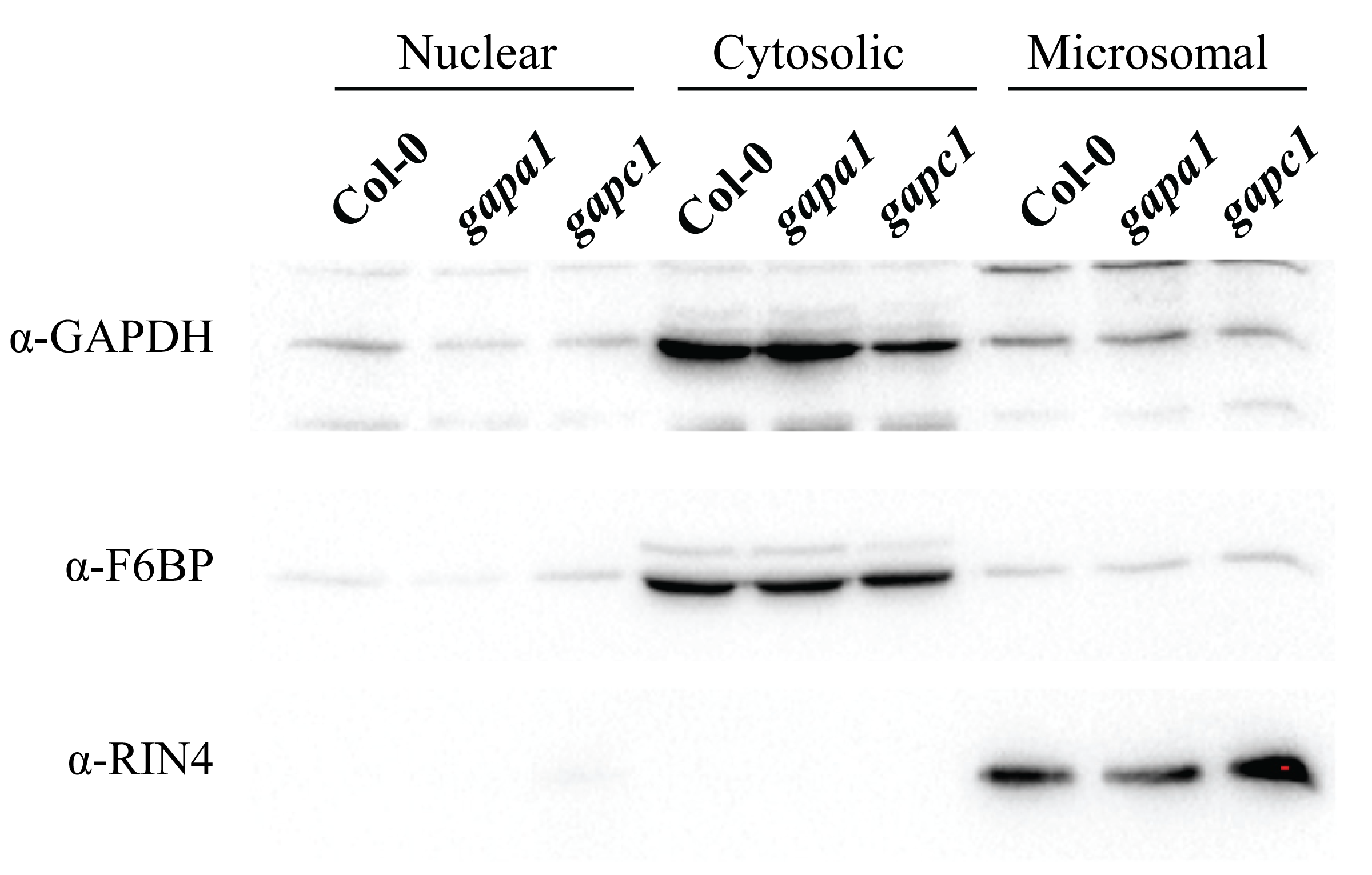

Supplement: S6 Fig — Nuclear, cytosolic and microsomal fractions from two-week-old seedlings were isolated using the Minute Plasma Membrane Protein Isolation kit (Invent biotechnologies, Inc). Western blotting using α-GAPDH demonstrates primarily cytosolic localization with some protein found in the microsomal fraction and less in the nuclear fraction. Western blotting with marker proteins was used to verify enrichment of individual fractions. α-F6BP is a cytosolic marker and α-RIN4 is a microsomal marker. A total of 8μg of protein was loaded per lane. (TIF) [file pgen.1005199.s006.tif]

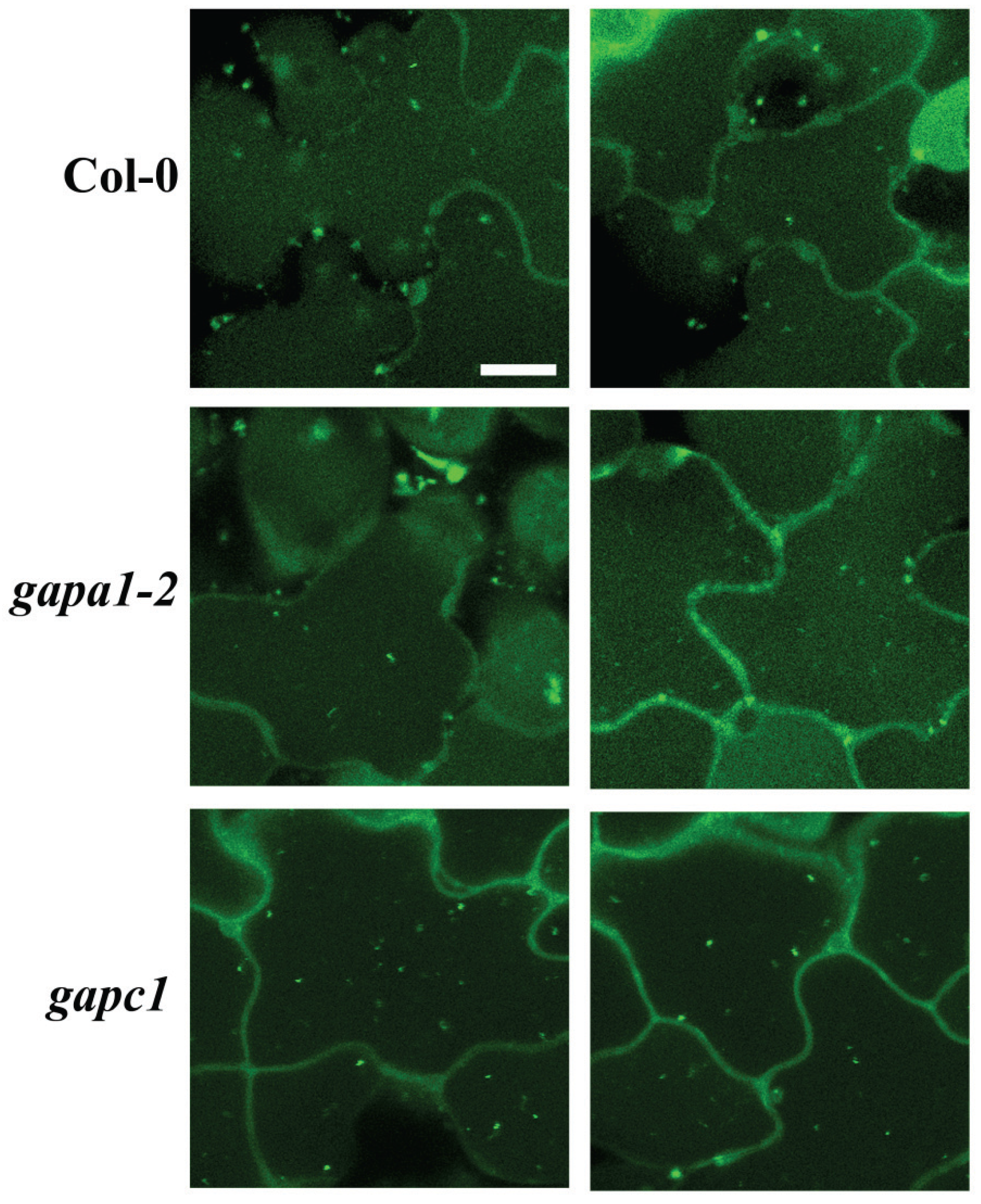

Supplement: S7 Fig — Two-week-old seedlings were grown on MS media without nitrogen for four days, and then incubated with the 50μM of the fluorescent dye MDC for 3 h. Autophagy bodies were visualized by confocal microscopy. Two independent images are shown for each genotype. Scale bar = 10 μm. (TIF) [file pgen.1005199.s007.tif]
