# Supplementary material for: Beyond Glycolysis: GAPDHs Are Multi-functional Enzymes Involved in Regulation of ROS, Autophagy, and Plant Immune Responses
Source: PLoS Genet. 2015 Apr 28;11(4):e1005199. doi: 10.1371/journal.pgen.1005199 (PMC4412566; doi:10.1371/journal.pgen.1005199)
Supplement: S1 Table — (5’ to 3’) (PDF) [file pgen.1005199.s008.pdf]

TABLES:

Table S1. Primers used in experiments.

At3g04120 R clone	GGCCTTTGACATGTGGAC
At3g04120 F clone	CACCATAGCAATTGACGGGAC
At3g04120 L seq	TCCCGTCGCTGCTATTTTAC
At3g04120 R seq	AACAACCTTCTTGGCACCAC
At3g26650LP1	GAGTTGCAGGATTTTGTCTGC
At3g26650RP1	CAGCATCAAAGATTCCGAGAG
At3g26650LP2	TGATAACCTTCTTGGCACCAG
At3g26650RP2	GTCAGACAAATGGAGAGCAGC
QRT-A1-F	CTCCCTTGGAAGGAGCTAGG
QRT-A1-R	TTCTTGGCACCAGCTTCAAT
A2-QRT-F	TAGATGCGAGCCACCGTGATCTAAGG
A2-QRT-R	GCACACGCAATGCAATTCCGTTAAG
GAPB-QRT-F	ACTCTTTCCCTGCTCAATGC
GAPB-QRT-R	GGAGTTGATGTTGTCACAGC
C1-QRT-F	CTTCCAGCTCTTAACGGAAAG
C1-QRT-R	GCCAACGAAGTCAGTTGAGACA
C2-QRT-F	AGTGTTGCCATCCCTCAATGGA
C2-QRT-R	AAGGTCAACGACACGAGAACTGT
Cp1-QRT-F	GTCGCAACCTGAAGCCATCAAG
Cp1-QRT-R	GTACTTGGCATCAATGAATGGG
Cp2-QRT-F	TCTTCCAATTCGACCAAAACC
Cp2-QRT-R	TCCTCGTGTCGAGCTTTATCC
AMV019-bZIP60	AGGACGTATGCTTGAGTGCTTCGT
AMV020-bZIP60	TTCTGGACGTAGGAGGCAACACT
Ef1a-4_Fwd	TGA GCA CGC TCT TCT TGC TTTCA
Ef1a-4_Rev	GGTGGTGGCATCCATCTTGTTACA
Atactin 2 F	GGAAAGGATCTGTACGGTACC
Atactin 2 R	TGTGAACGATTCCTGGAC
EF1a qPCR F	CTGGATTCGAGGGAGACAACA
EF1a qPCR R	GCACCGTTCCAATACCACCAA
PR1 F	CGGAGCTACGCAGAACAACT
PR1 R	CTCGCTAACCCACATGTTCA
